# RaspyControl Lab: A fully open-source and real-time remote laboratory for education in automatic control systems using Raspberry Pi and Python

**DOI:** 10.1016/j.ohx.2023.e00396

**Published:** 2023-01-13

**Authors:** Jonathan Álvarez Ariza, Christian Nomesqui Galvis

**Affiliations:** Department of Technology in Electronics, Engineering Faculty, Corporación Universitaria Minuto de Dios-UNIMINUTO, 111021 Bogotá, Colombia

**Keywords:** Remote laboratory, Automatic control, Engineering education, Open-source hardware, Open-source software, Raspberry Pi, Python, Control systems

## Abstract

Currently, remote laboratories have gained relevance in engineering education as tools to support active learning, experimentation, and motivation of students. Nonetheless, the costs and issues regarding their implementation and deployment limit the access of the students and educators to their advantages and features such as technical and educational. In this line, this study describes a fully open-source remote laboratory in hardware and software for education in automatic control systems employing Raspberry Pi and Python language with an approximate cost of USD 461. Even, by changing some components, the cost can be reduced to USD 420 or less. To illustrate the functionalities of the laboratory, we proposed a low-cost tank control system with its respective instrumentation, signal conditioning, identification, and control, which are exposed in this document. However, other experiments can be easily scalable and adaptable to the remote laboratory. Concerning the interface of the laboratory, we designed a complete user-friendly web interface with real-time video for the users to perform the different activities in automatic control such as identification or controller implementation through the programming language Python. The instructions to build and replicate the hardware and software are indicated in the open repositories provided for the project as well as in this paper. Our intention with this project is to offer a complete low-cost and open-source remote laboratory that can be adapted and used for the students, educators, and stakeholders to learn, experiment, and teach in the field of automatic control systems.

## Graphical abstract

**Figure:**
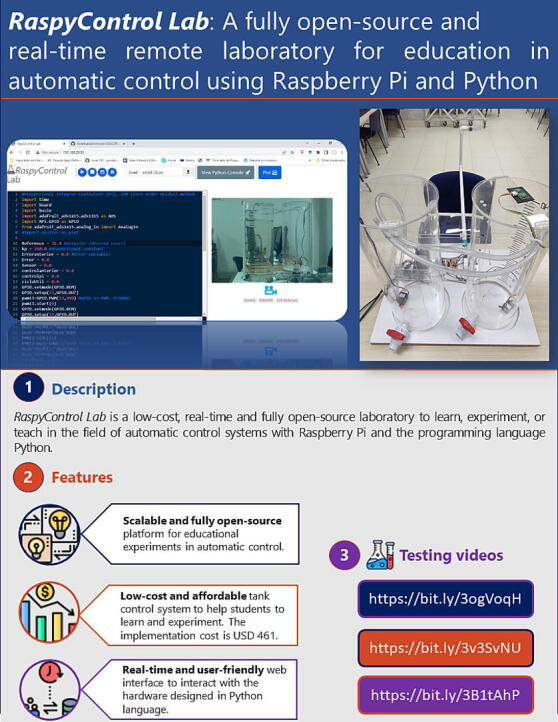

**Specifications table:**

**Table:** 

**Hardware name**	*RaspyControl Lab*
**Subject name**	• Educational tools and open source alternatives to existing infrastructure • Measuring physical properties and in-lab sensors • Field measurements and sensors • Electrical engineering and computer science
**Closest analog**	**UniLabs**: https://unilabs.dia.uned.es/
**Open source license**	Creative Commons Attribution-ShareAlike 4.0 International (CC BY-SA 4.0)
**Cost of hardware**	⩽USD 461
**Source file repository**	• **Hardware:** https://doi.org/10.5281/zenodo.7500242 • **Software:** https://doi.org/10.5281/zenodo.7500242 https://github.com/Uniminutoarduino/RaspyControlLab

## 1 Hardware in context

During the last two decades, Remote Laboratories (RLs) have contributed to enhancing the process of teaching–learning, primarily in engineering. Several factors such as the increase in the number of students in the classrooms, the need to provide high-quality technological tools for the students to learn and experiment, and the reduction of the economic resources destined for physical infrastructure have led to this situation [1], [2]. In this way, laboratories represent the basis of the experimentation in engineering and computer science and they have formed an active part of the curricula in these disciplines. Students learn and experiment with hands-on activities, which are the essence of science learning [3], [4], [2]. Several architectures and long-term RLs have been created under the previous principles with successful examples such as the open architecture known as Virtual Instrument Systems in Reality (VISIR) [5], [6], [7], [8] widely spread through the cooperation of several universities or the RL WebLab-Deusto LabsLand [9], [10]. In the context of open-source RLs, the study of Lavayssière et al. [11], [12] exposes an open-source RL for electronics called *Laborem Box* with Raspberry Pi. The authors indicate that there are several RLs such as UNILabs [13], RexLab [14], GOLDi [15], Farlabs [16], among others, that are not open-hardware while this study is. The solution has a cost of USD 429.35. Besides, the work [17] presents low-cost experiments in the field of automatic control, which are described with the perception of *n* = 250 students who used them in a control and instrumentation course. By the same token, Sáenz *et al*. [18] show the RLs for Multiple-Input and Multiple-Output (MIMO) control systems at the National University of Distance Education (UNED) in Spain. The authors expose the architecture of the RL and the identification of the control plant with its respective controller and step response. Another architecture of RLs for embedded systems is described in [19], where the authors depict that the RL counts with both an interactive live-streaming platform and a Remote Laboratory Management System (RLMS). In addition, the authors show the perception of *n* = 27 students with their comments. Complementary, some organizations such as the Global Online Laboratory Consortium (GOLC) [20] have disseminated the studies and advances about remote laboratories for industrial and academic purposes.

Although the previous studies and technologies orientated the aspects considered in the design, construction, and deployment of the *RaspyControl Lab*, we intended to complement these studies in the sense to provide a fully open-source laboratory for automatic control with Raspberry Pi and Python that can be adapted to the technical and educational requirements of educators and practitioners. *RaspyControl Lab* started with some prior studies and curricular reflection of the research group in engineering education at the Corporación Universitaria Minuto de Dios-UNIMINUTO [21], [22] with the help of students before and during the COVID-19 pandemic. These works were oriented to provide high-quality and low-cost learning tools for our students to experiment with hands-on activities in the areas of circuits, signal processing, and physical computing due to the lack of low-cost RLs that could be easily replicable and deployed. In special, *RaspyLab* [21] has been tested with around *n* = 90 students of engineering and computer science in the topics concerning physical computing and programming. Also, since several of the previous RLs are open-source but employ, for instance, infrastructures such as application servers, or National Instruments (NI) data and signal acquisition units [23] that result expensive, we decided to construct our low-cost hardware and open-source software to support the different tasks of learning and experimentation of the students in automatic control. Table (1) shows a brief synthesis of the most relevant RLs in comparison with our work. Other complementary alternatives can be found in the references [17], [24], [25].

**Table 1:** Description of hardware and software of the main RLs in comparison with *RaspyControl Lab*.

**Remote Laboratory**	**Reference**	**Open source hardware**	**Open source software**
**1.** Laborem	[11], [12]	Yes	Yes
**2.** UNILabs	[13]	No	Yes
**3.** WebLab-Deusto LabsLand	[9], [10]	No	Yes
**4.** Farlabs	[16]	No	No
**5.** RexLab	[14]	No	Yes
**6. RaspyControl Lab (own)**	[26], [27]	Yes	Yes

**Fig. 1:**
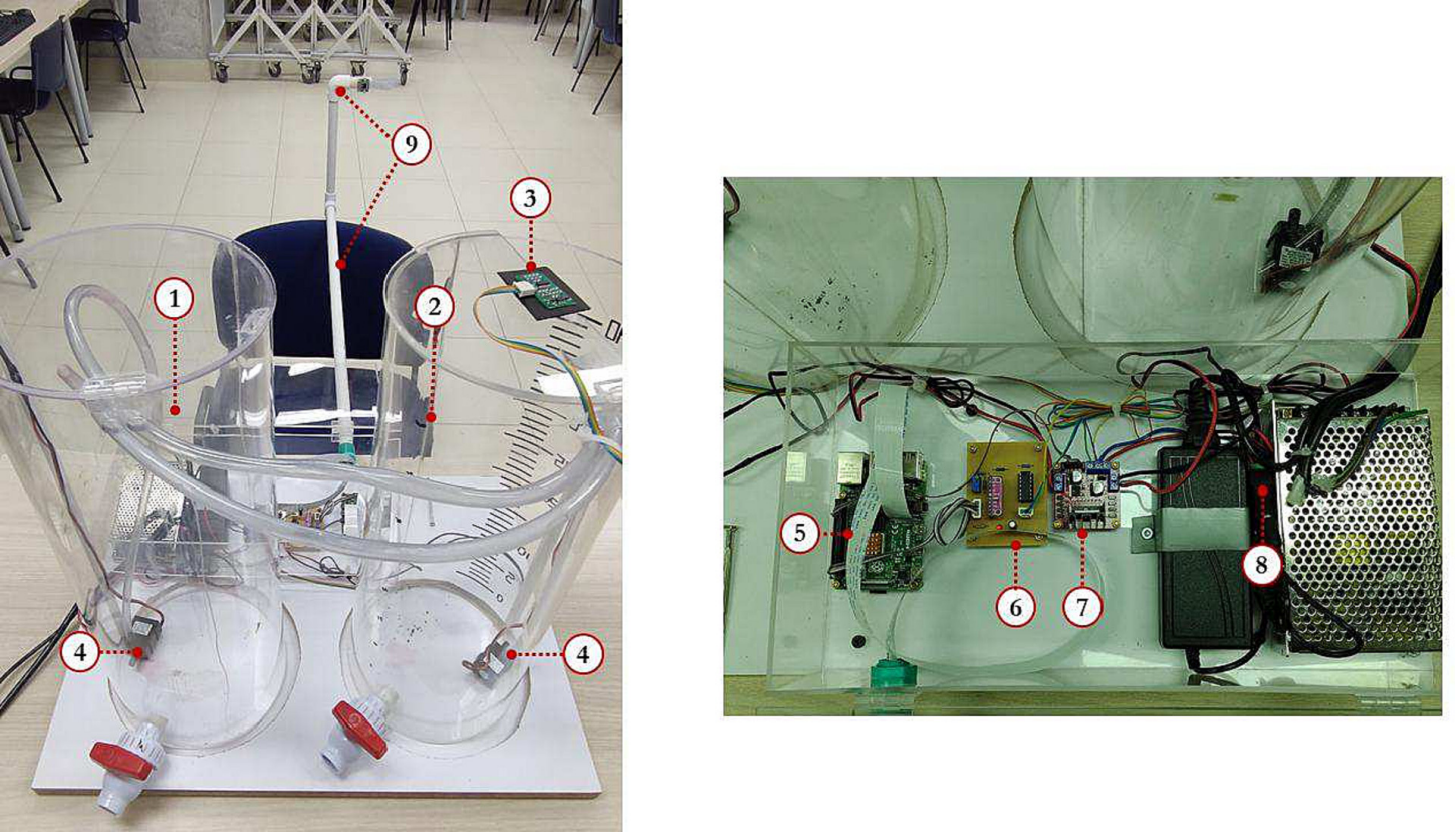
Overall hardware appearance of *RaspyControl Lab*. 1. Reservoir tank (T2), 2. Main control tank (T1), 3. Analog ultrasonic sensor, 4. 12 V motor pumps, 5. Raspberry Pi 4, 6. Signal conditioning circuit with ADC (ADS1115), 7. L298 motor pump driver, 8. 12 V and 5 V Power supply sources, 9. Raspberry pi camera with PVC pipe support.

One purpose of this project is that the designed hardware can be easily constructed and replicated with accessible materials and components with local or international distributors. Likewise, hardware is easy to understand in theircomponents such as the conditioning circuit for the ultrasonic sensor and the 16-bit Analog-to-Digital Converter (ADC) ADS1115, the motor driver with Pulse Width Modulation (PWM) for the 12 V immersion motor pumps, or the connections with the Raspberry Pi. Thus, this paper describes the open-source hardware and software designed and implemented for the RL *RaspyControl Lab*. To test the RL, we designed and implemented a low-cost tank control system with its signal conditioning for the Raspberry Pi. Then, the users or students can control the level *h* of the main tank (T1). To illustrate the hardware design, Fig. (1) shows its overall structure.

The remainder of the paper is divided as follows: Section 2 depicts the hardware composed of the control tanks and the signal conditioning circuit with its respective Printed Circuit Board (PCB). Section 3 describes the open software created with a special emphasis on real-time video and the web interface for the users. Section 4 shows the list of design files for software and hardware. Section 5 describes the Bill of Materials (BOM). Sections 6, 7 show the build and operation instructions for the hardware designed, including a troubleshooting subsection. Section 8 exposes the validation of the RL with the different experiments for the identification and control of the plant. Finally, Section 9 outlines the conclusions and further work of this study.

## 2 Hardware description

The main features and advantages of our hardware for educators and students are the following:

• Scalable and fully open-source platform for educational experiments in automatic control.
• Low-cost and affordable tank control system to help students to learn and experiment in the field of automatic control systems.
• User-friendly web interface with real-time video to interact with the hardware designed in Python language.
• Simple, low-cost, and low-power conditioning circuit for the ultrasonic sensor and ADC (ADS1115) with a maximum of 860 Samples Per Second (SPS).

Before starting with the description of hardware and software components, it is important to mention that we employed the typical convention of control systems in the Laplace (*s*) and *z* transform domains to designate the hardware as follows:

• G(s),G(z): Control plant: Main tank (T1). The variable to control is the level (*h*).
• H(s),H(z): Analog ultrasonic sensor with its signal conditioning circuit.
• C(s),C(z): Controller (P, PI, or PID).
• R(s),R(z): Setpoint (level reference). Y(s),Y(z): Control output for the tank (T1)-level (*h*).
• E(s),E(z): Error.

### 2.1 Overview

**Fig. 2:**
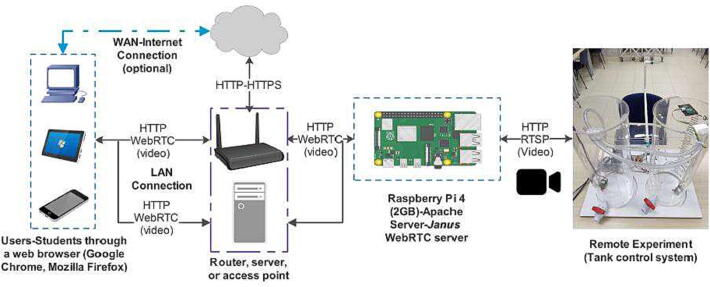
Network scheme for *RaspyControl* Lab.

Fig. (2) depicts the overall architecture of *RaspyControl Lab*. In this case, the hardware consists of the remote experiment that is controlled through a Raspberry Pi 4. To achieve this remote control, the Raspberry Pi has an Apache server installed with both HTTP protocol and the complement known as Web Server Gateway Interface (WSGI) to interact with Python language. The Apache server allows access to the RL and contains the web pages for the students to control the experiment, employing a PC, laptop, tablet, or smartphone. The real-time video of the experiment is encoded from a Raspberry Pi camera using the tool *FFmpeg* [28] that sends the video frames, which are encapsulated into the Real-time Streaming Transport Protocol (RTSP). The frames are sent to a special open-source and real-time video server installed in the Raspberry Pi known as *Janus WebRTC* [29], [30] that transforms the frames in RTSP protocol towards the standard WebRTC, compatible with browsers such as Google Chrome or Mozilla Firefox. As a concept, Web-based Real-Time Communication (WebRTC) [31], [32] is a relatively new standard for real-time peer-to-peer communications that has several advantages for gaming, video streaming, and sensor data feeds.

When the students want to perform identification or control of the plant, they must construct an algorithm in Python language that on one hand, reads the analog value of the ultrasonic sensor (US-016) through the ADC (ADS1115) to identify the current level of the tank, and on the other hand, manages the motor pumps of the tanks with the General-Purpose I/Os (GPIOs) of the Raspberry Pi. The ADC is interfaced to the Raspberry Pi with the protocol $I^{2}C$. After, the algorithm is transferred to the Apache server in the Raspberry Pi for further processing. For the hardware described in this paper, we employed a Local Area Network (LAN) with the scheme of Fig. (2). However, remote access to the experiment could be guaranteed from the same Raspberry Pi with its Domain Name System (DNS) or employing a Learning Management System (LMS) as Moodle with few modifications in the software. We wanted to leave this choice according to the technical requirements of the educators or stakeholders interested in the project. Nonetheless, some suggestions for the Internet access of the experiment are indicated in Section 7.1. To extend the hardware features, each one of its components will be explained in the next subsections.

### 2.2 Control tank system

The control tank system is composed of two cylindrical acrylic tanks (*height (h)* = 51 cm, *diameter(dia)* = 20.8 cm, *thickness* = 4 mm) and two (12 V, 4.8 W) immersion motor pumps with a maximum flow and impulse height of ($Q_{\max}$ = 240 L/H, $h_{\max}$ = 300 cm), respectively. One acrylic tank (T1) is the control plant G(s) with its ultrasonic sensor and conditioning H(s), while the tank (T2) serves as a reservoir. Both tanks count with motor pumps to interchange liquid between them, which are interfaced with flexible plastic hoses. Concerning the ultrasonic sensor (US-016), this is an analog sensor with a measurement range between 2 cm to 300 cm and a resolution of 1 mm. For 1 meter measurement, the formula of sensed distance vs. output voltage of the sensor is given by the expression $h(\mathrm{mm})=1024\;*\;\frac{V_{\mathrm{out}}}{V_{\mathrm{cc}}}$, where *h* is the tank level in mm, and $V_{\mathrm{cc}}=5V$.

**Fig. 3:**
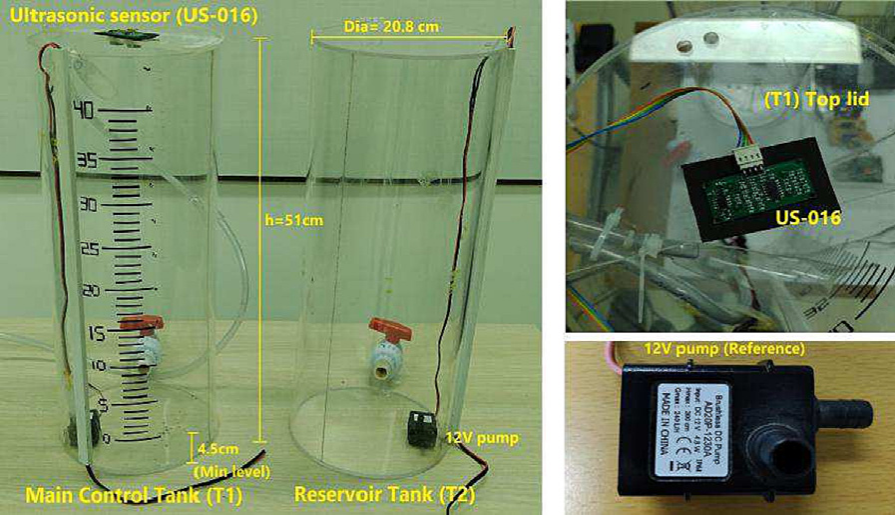
Control tank system for *RaspyControl* Lab.

Fig. (3) shows the overall appearance of the control tank system. Due to the tanks were created by a local manufacturer, we designed the different CAD files in TinkerCAD that are available in the section to design files to aid educators and practitioners in their construction in an easy form.

### 2.3 Signal conditioning circuit

**Fig. 4:**
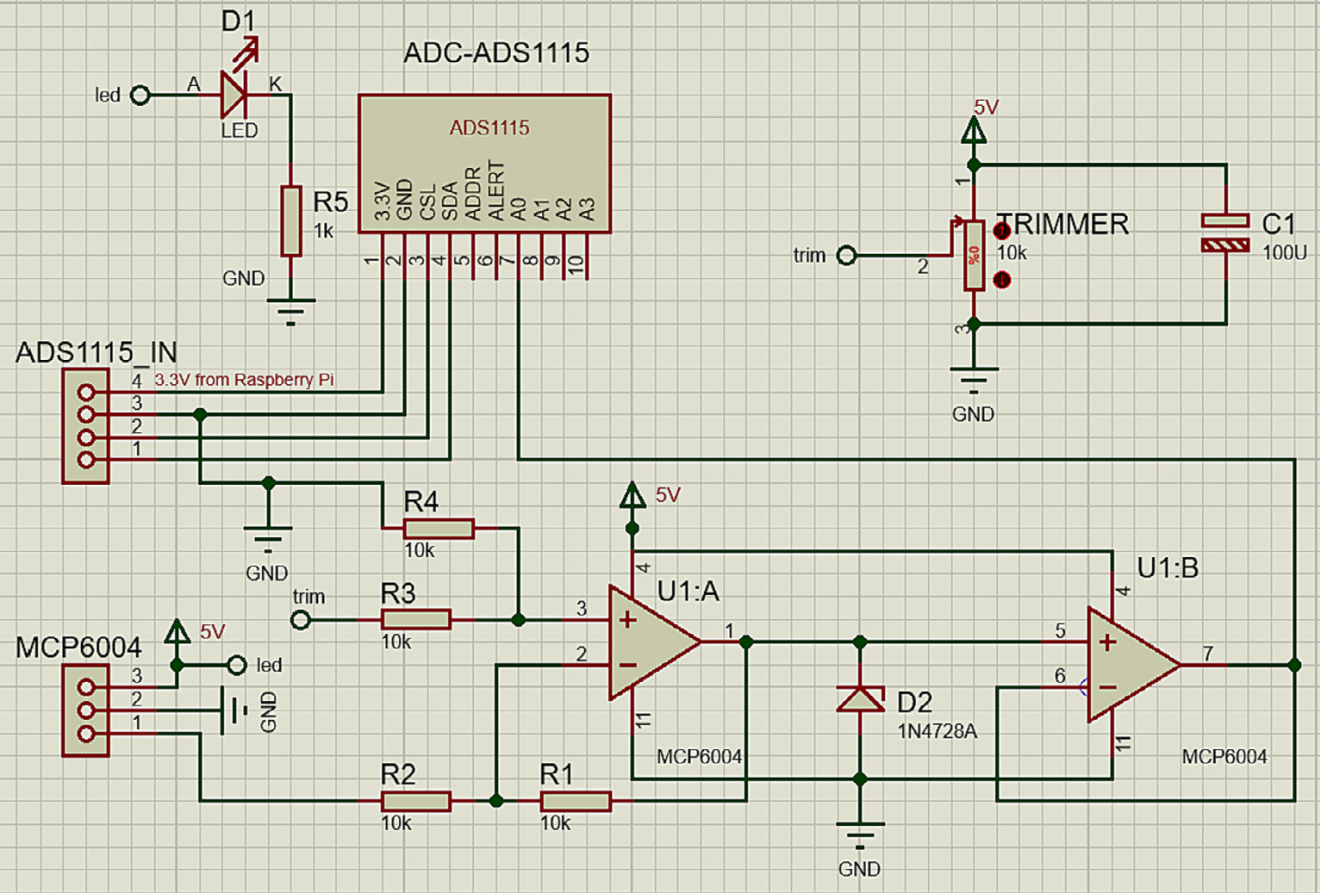
Schematic of the signal conditioning circuit for *RaspyControl* Lab.

The purpose of the signal conditioning circuit is twofold. On one hand, it allows calibrating the minimum level of detection of the control tank (T1) with the ultrasonic sensor. Because the immersion pumps need a minimum level of liquid to prevent damage, we set this minimum level with a value of **4.5 cm over the base of the tanks** (T1,T2) as Fig. (3) depicts. Therefore, the signal conditioning circuit guarantees that to this level, its output is 0 V. On the other hand, the circuit helps to reduce the noise from the sensor because of the Common-Mode Rejection Ratio (Typical CMRR = 76 dB) of the MCP6004. The conditioning circuit is composed of a subtracter made with the operational amplifier (MCP6004), some resistors, and a 10KΩ trimmer as Fig. (4) illustrates.

Since the resistor values of the subtracter are 10KΩ ($R_{1}-R_{4}$), the output voltage for this circuit is $V_{\mathrm{out}}=V_{\mathrm{OFF}}-V_{\mathrm{sens}}$, where $V_{\mathrm{OFF}}$ is the adjust voltage with the trimmer, and $V_{\mathrm{sens}}$ is the output voltage of the ultrasonic sensor. Nonetheless, if a gain is required for this circuit, the resistor $R_{1}$ could be changed. The output voltage generated by the subtracter circuit is directly wired to channel A0 of the ADS1115, even though this device counts with 4 channels bounded in the range 0 V-3.3 V. The only precaution is not to exceed the 3.3 V for the input (channel A0) of the ADS1115. This can be done with a 3.3 V zener diode such as 1N4728A (D2 in schematic), acting as a limiter in the output of the MCP6004. In the experiment, the 3.3 V value is not exceeded due to the tank height (*h*) and the voltage output of the ultrasonic sensor. In this case, the control experiment for *RaspyControl Lab* is a Single-Input Single-Output (SISO) system because only a variable (level) is controlled. However, if new variables need to be added for Multiple-input Multiple-output (MIMO) systems, the circuit can be redesigned to meet these specifications.

**Fig. 5:**
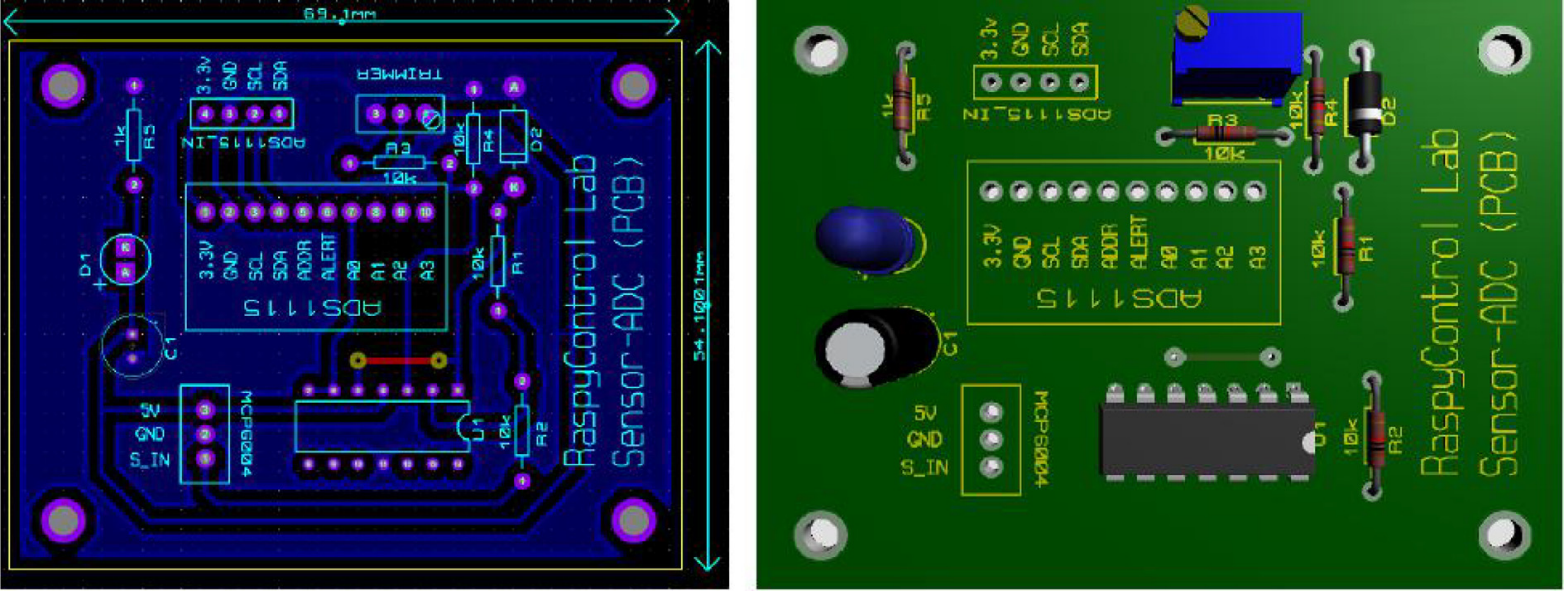
PCB designed for the signal conditioning circuit.

**Fig. 6:**
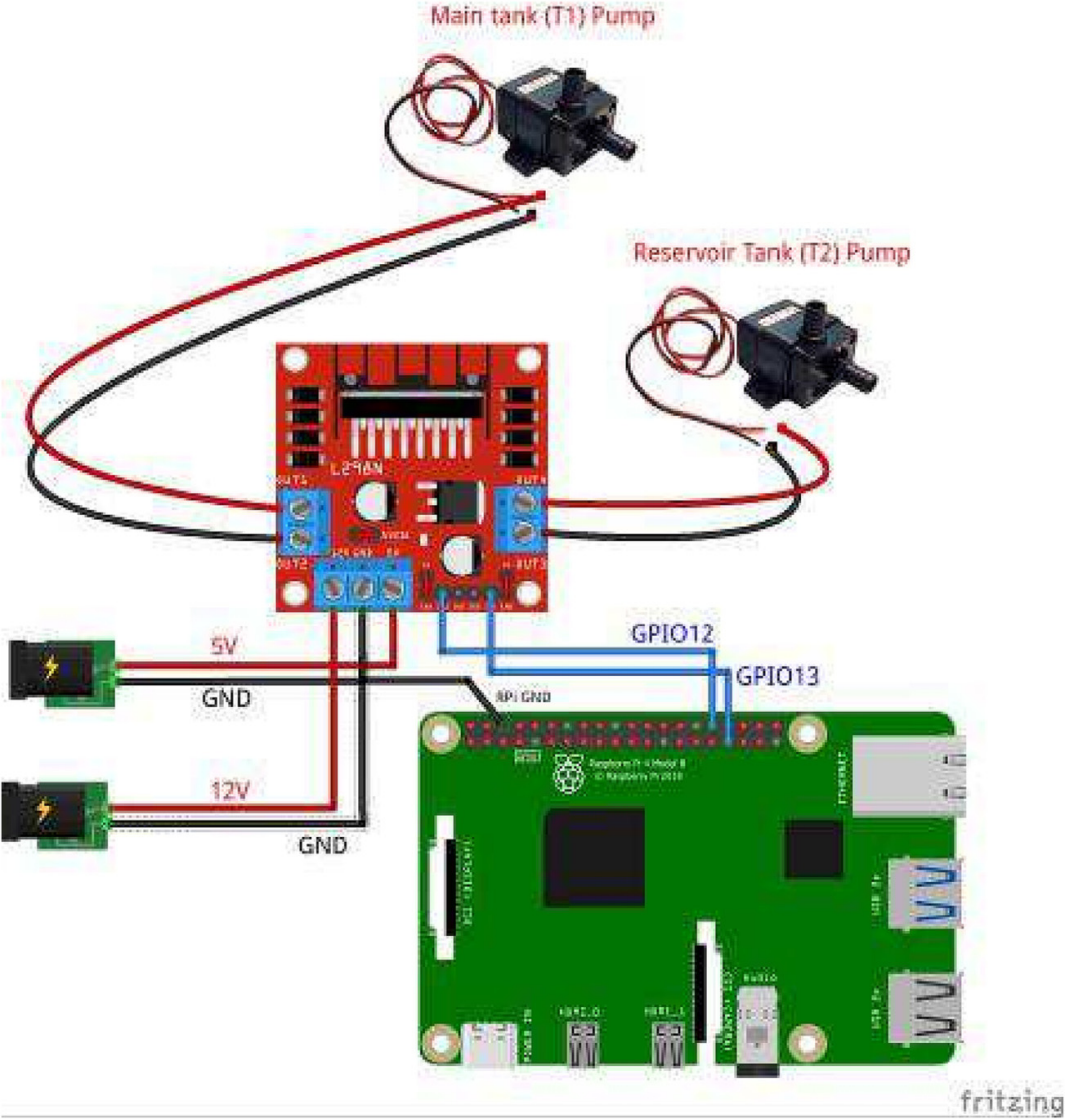
Schematic for the L298 driver and Raspberry Pi. In this case, the ground of the 5 V and 12 V power supplies is wired with the ground of the Raspberry Pi 4.

**Fig. 7:**
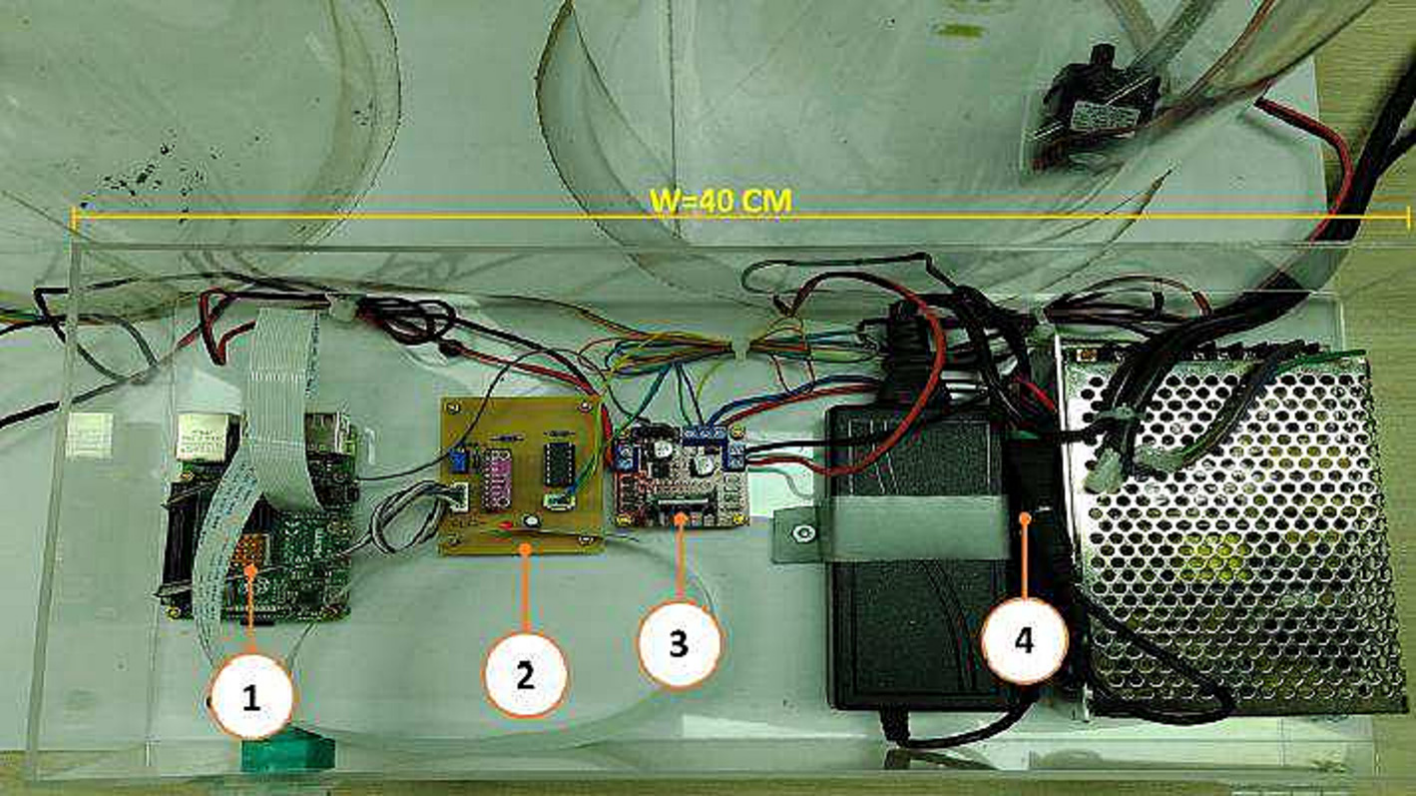
Components’ box of *RaspyControl* Lab. 1. Raspberry Pi 4 (4 GB), 2. Signal conditioning circuit, 3. L298 motor pumps driver, 4. Power supplies (5 V, 12 V).

**Fig. 8:**
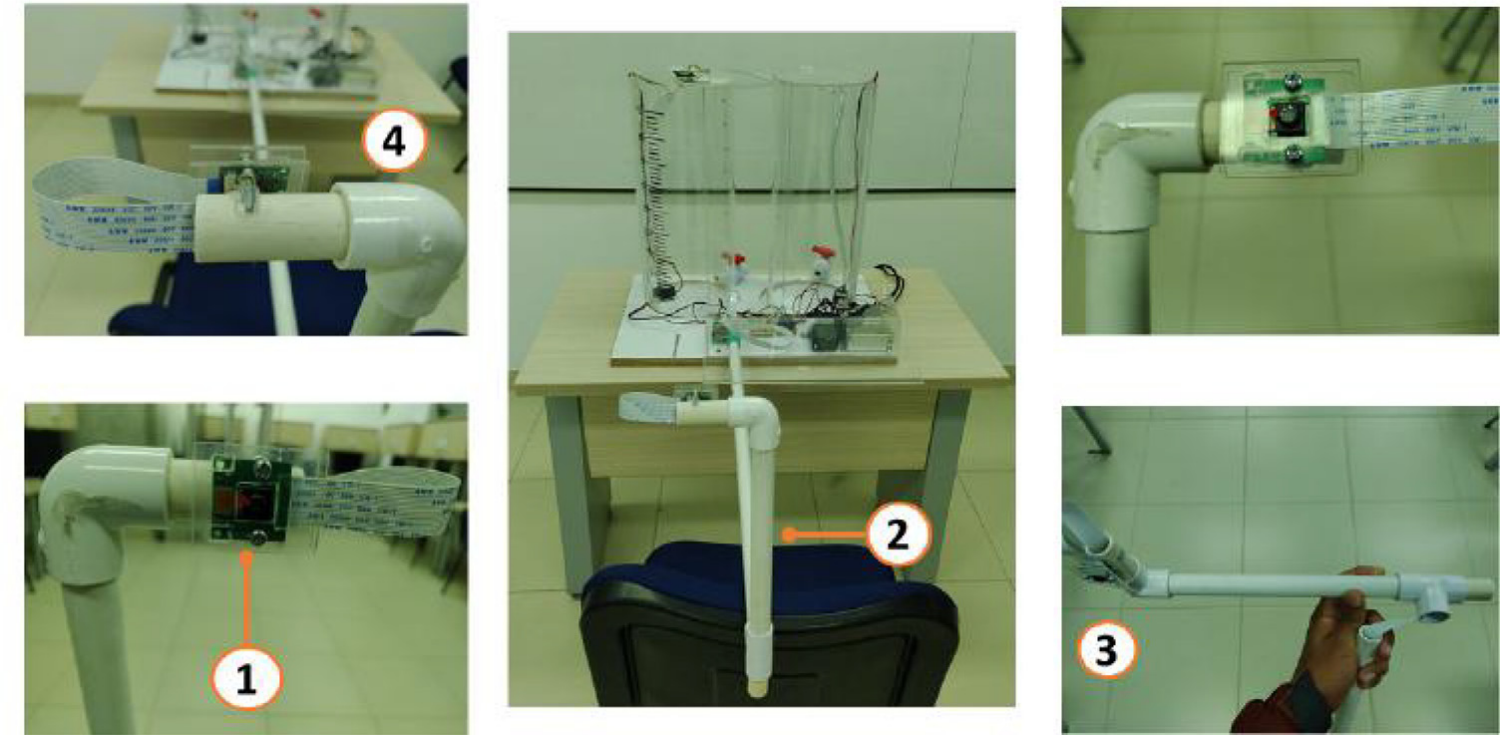
Camera support for the experiment. 1. 8MP camera. 2. Vertical PVC pipe segment. 3. Flex cable inside 0.5-inch PVC pipe. 4. Rounded PVC coupling to adapt the camera connection.

The circuit has two power supply options. The MCP6004 and sensor operate with 5 V, whereas the ADS1115 with a 3.3 V. Hence, we separated the power supplies for the signal conditioning circuit and the motor pumps in order to reduce electric noise levels in the system. Besides, the ADS1115 was directly interfaced to 3.3 V and the pins SCL and SDA for the $I^{2}C$ protocol in the Raspberry Pi. This design selection was due to the ultrasonic sensor (US-016) is a sensible device whose measurement could be affected by ripple voltages or electric noise. Thus, we employed two switched-mode power supplies as Fig. (6) and Fig. (7) show due to their low ripple voltage (±0.3V). The power supplies must be attached to the connectors (MCP6004 and $\mathrm{ADS}1115_{\mathrm{IN}}$) according to the schematic of Fig. (4). Besides, the input $S_{\mathrm{IN}}$ in the connector MCP6004 allows to interface the sensor voltage output. With these aspects, we designed a Printed Circuit Board (PCB), which is outlined in Fig. (5) with the dimensions (*w* = 6.91 cm, *h* = 5.41 cm). Both the schematic and the PCB were designed in the software Proteus VSM 8.9. (See Fig. (8)).

Regarding the 16-bit ADC (ADS1115), this is an $I^{2}C$ ADC with four analog channels to convert, and a Programmable Gain Amplifier (PGA). The ADC1115 is attached to the 3.3 V power supply of the Raspberry Pi as mentioned. The programmable data rate for this device goes from 85 SPS to 860 SPS, which is ideal for the control plant because it is not a fast system. Indeed, as we will see in the section of validation and characterization, the settling time ($t_{s}$) of the control plant for h=40cm is ($t_{s}\approx 262\mathrm{secs}$). Data from the ADS1115 is read in terms of voltage, employing the Adafruit Python Library (circuitpython adafruit_ads1x15 [33]).

### 2.4 Motor pumps power driver

Each motor pump is controlled through a GPIO (12, 13) in the Raspberry Pi with PWM support. The selected PWM frequency was $f_{\mathrm{pwm}}=490\mathrm{Hz}$. The maximum current consumption of each motor pump with a 12 V power supply is I≈0.4A, and the power consumption is (P=4.8W), which is ideal for the nominal capacity of the motor driver L298 (P=20W) that is used in the control system. The Duty Cycle (*k*) of each PWM signal is changed with the controller created by the students. Specifically, the students should use the Python library **RPi.GPIO** with the following methods, where **pwm13** is an object for the pin (GPIO 13) to access the different functions of the library RPi.GPIO:

**Table:** 

pwm13 = GPIO.PWM(13,490)
pwm13.ChangeDutyCycle(50)

The first method configures the pin and frequency, while the second changes the duty cycle *k* in percentage (0–100%). Fig. (6) shows the overall schematic with the motor pump driver (L298) and the Raspberry Pi 4.

### 2.5 Components’ Box and camera support

Fig. (7) shows the component box of *RaspyControl* Lab with its components. In the box are allocated the main hardware components of the control system such as the Raspberry Pi, motor pump driver, signal conditioning circuit, and power supplies. The component box was constructed in acrylic with the following dimensions: (h=9.8cm,l=20cm, and w=40cm).

The components were attached to the box with screws type M3 and M2.5 with their nuts. Regarding the camera, this is an 8MP Raspberry Pi camera interfaced with a flex cable of 2 m. The flex cable was put inside a 0.5-inch PVC pipe with a length of l≈1.2m, including the vertical segment in which the camera is installed as Fig. (1) depicts. We selected this distance to provide a good video focus on the experiment.

### 2.6 3D model

**Fig. 9:**
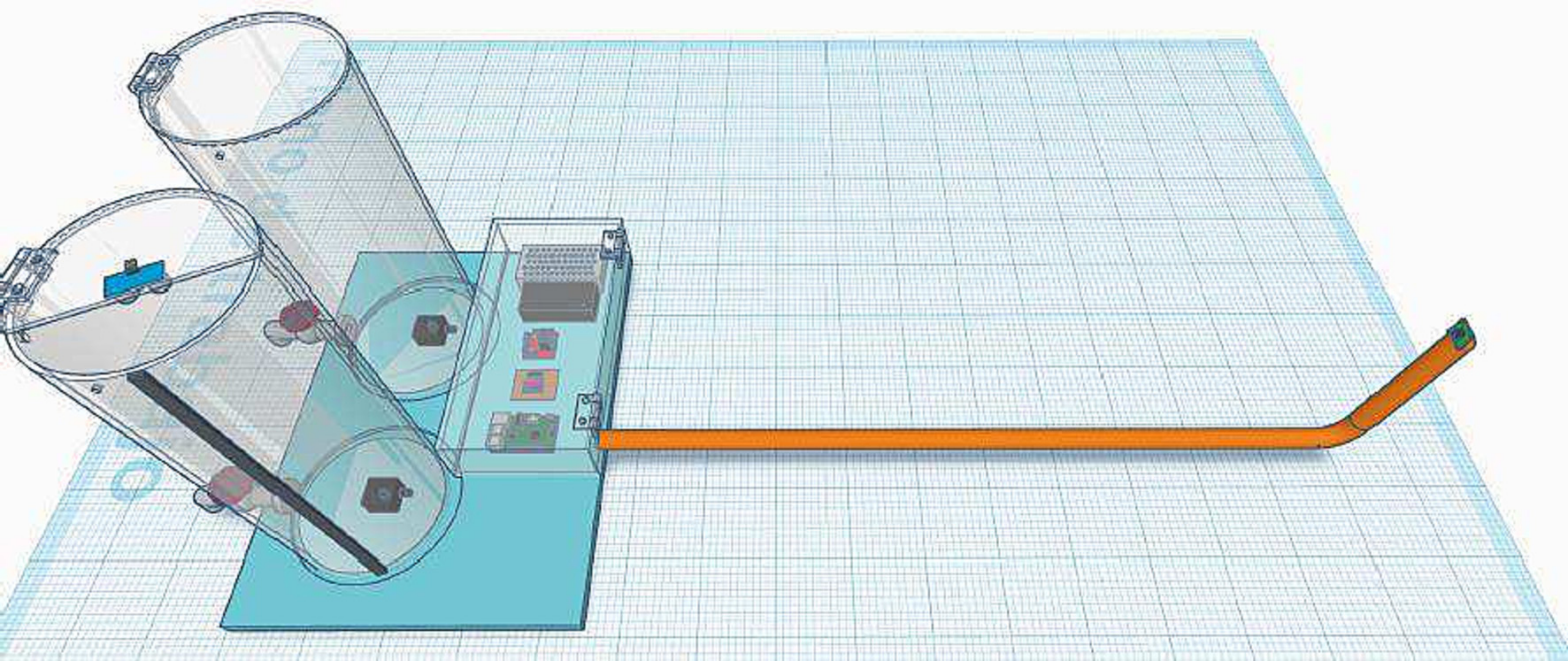
TinkerCAD 3D model of *RaspyControl* Lab.

As described, to facilitate the construction of the tanks, and component’s box, and to offer a glance at the component distribution, we created an approximate 3D model of the hardware with all its dimensions in the software TinkerCAD. The link with the model is available in Section 4. Fig. (9) illustrates the model. Bear in mind that the dimensions of the model are in scale 1:10.

## 3 Software description

**Fig. 10:**
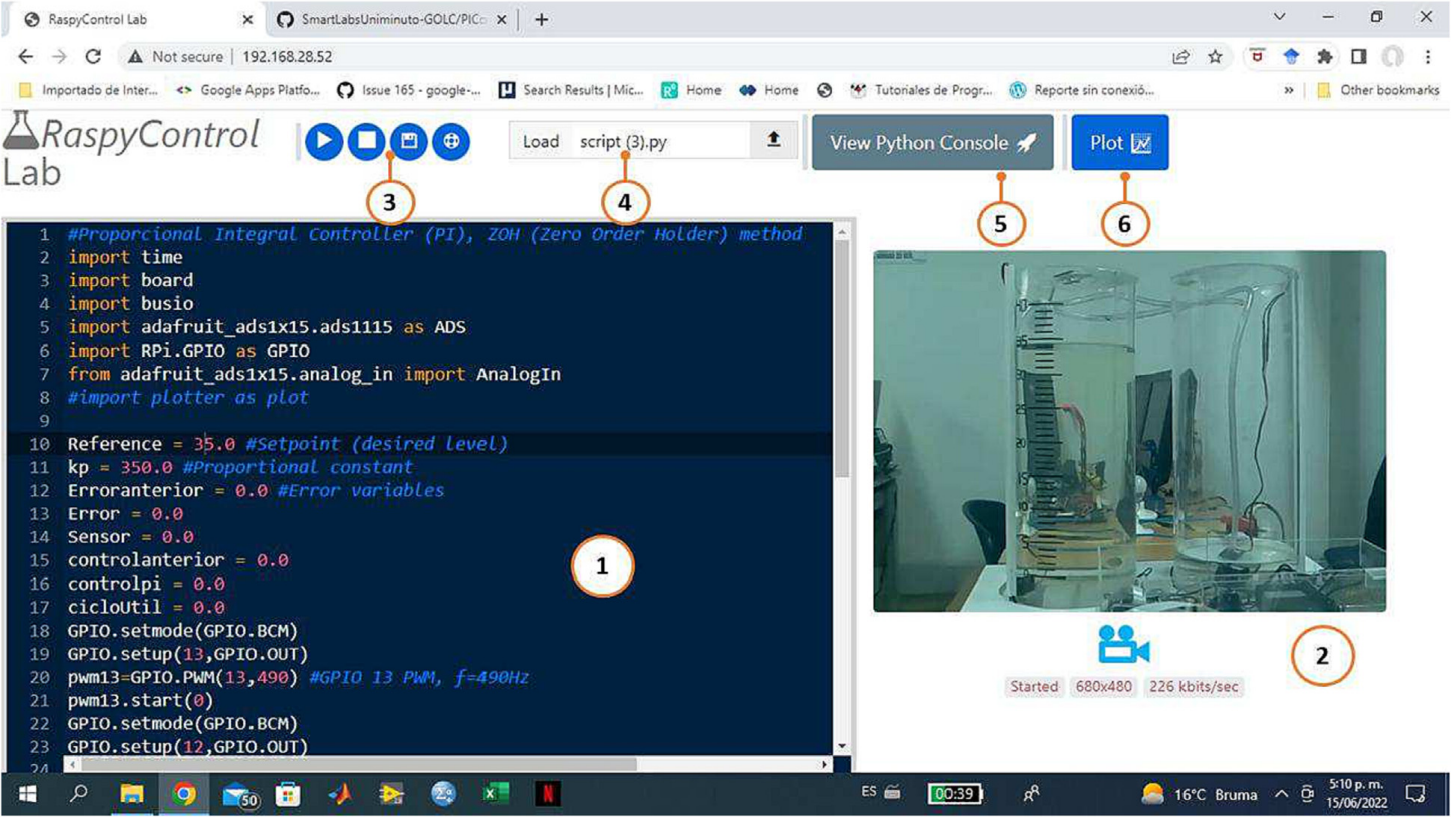
User-friendly Web interface for *RaspyControl* Lab. 1. Working area, 2. Real-time video, 3. Buttons for the functions of help, execute, stop, and save script, 4. Load script form, 5. Button to view Python Console, 6. Button to plot data from the script.

The software component of *RaspyControl* Lab is composed of a web interface in which the students and educators can identify the control plant, test the controllers, and easily debug them. The interface has the elements depicted in Fig. (10). In the working area, the students or educators can construct their control algorithms in Python to perform different actions in automatic control such as identification, and both testing and debugging of the controllers. At any moment, the users can see the control plant behavior with the real-time video streamed from the camera installed in the experiment. The video frames encoded in the WebRTC standard through the Janus WebRTC server are shown in the user interface with a maximum bandwidth of 200 Kb/s. The developers or educators are encouraged to change this bandwidth with the instructions of the software repository at any moment according to the features of the network in which the experiment is mounted. The latency experimented in the different experiments that we made, oscillated between 500 ms to 3 s.

When the students finish their algorithms in the working area, they can click on the run button to execute the Python script in the experiment. Likewise, the students or educators can save or upload the scripts created in the interface. At last, the buttons *view Python console* and *plot* allow the students to see the Python console of the Raspberry Pi to check errors in the code execution or print information, and plot data of the Python script from the sensor, controller, etc., respectively. The web interface is accessed from a web browser compatible with the WebRTC standard such as Google Chrome or Mozilla Firefox. Only the users must type the IP of the Raspberry Pi in the experiment through the browser, for instance,  http://192.162.5.2, or with the domain name  http://raspberrypi. All information of the students’ scripts in Python is managed through an Apache Server with the mode WSGI using the HTTP port (80) in the Raspberry Pi. The web interface sends the created Python code by means of a POST query to the Apache server.

Thus, six key open-source library components were used in the creation of the software interface, which are described in Table 2. The Janus WebRTC Server was employed for the real-time video, NodeJS to plot the data of the scripts in real-time with a *redis* database, Flask to process the scripts created by the students in Python through the HTTP protocol, and at last, ACE code editor was utilized to highlight and edit the statements of the scripts in Python directly in the web interface.

**Table 2:** Main complements and frameworks employed in the software of *RaspyControl Lab*.

**Software complement or framework**	**Description**	**Link**
**1. Janus WebRTC Server**	Open-source, general purpose, WebRTC server designed and developed by Meetecho.	https://github.com/meetecho/janus-gateway
**2.NodeJS**	JavaScript runtime built on Chrome’s V8 JavaScript engine. Node.js is designed to build scalable network applications.	https://nodejs.org/en/
**3.Flask**	Web framework, Python module that lets you develop web applications easily.	https://flask.palletsprojects.com/en/2.1.x/
**4. ACE code editor and highlighter**	Embeddable code editor written in JavaScript. It matches the features and performance of native editors such as Sublime, Vim and TextMate.	https://ace.c9.io/
**5. Apache Server**	Open-source HTTP server for modern operating systems including UNIX and Windows.	https://httpd.apache.org/
**6. Redis**	Open-source (BSD licensed), in-memory data structure store, used as a database, cache, and message broker.	https://redis.io/

In addition to the previous software components, the instructions for developers that want to replicate the software from scratch are available in the GitHub repository ( https://github.com/Uniminutoarduino/RaspyControlLab). In the same way, the instructions are available in Sections 6, 7 for educators that only want to start the experiment with the components of hardware and software exposed in this document. Finally, the complete Raspberry Pi OS image ready to be downloaded is available in Section 4 with all software components developed in the study.

## 4 Design files

### 4.1 Design files summary

All the design files are available in the repository (https://doi.org/10.5281/zenodo.7500242). The datasets with the different experiments, including the plant identification and the controllers’ implementation are available in the same repository.

**Table:** 

**Design filename**	**File type**	**Open source license**
*ProteusVSM.zip*	CAD file with the schematic and PCB for the signal conditioning circuit in Proteus VSM 8.9.	Creative Commons Attribution-ShareAlike 4.0 International License (CC BY-SA 4.0)
*3Dmodel.zip*	URL to the TinkerCAD 3D model of the hardware designed and implemented.	Creative Commons Attribution-ShareAlike 4.0 International License (CC BY-SA 4.0)
*RaspberryPiOSImage2022-09–08.rar*	Complete image with the Raspberry Pi OS, and all software components installed or developed in the project.	Creative Commons Attribution-ShareAlike 4.0 International License (CC BY-SA 4.0)
*Scripts.zip*	Python v3.9 scripts with the different controllers (P, PI, PID) designed and implemented.	Creative Commons Attribution-ShareAlike 4.0 International License (CC BY-SA 4.0)
*MATLAB.zip*	MATLAB R2018b files with the simulations and SISO tool sessions created in the project.	Creative Commons Attribution-ShareAlike 4.0 International License (CC BY-SA 4.0)
*Dataset (identification-control).xlsx*	Datasets with the different experiments from the plant identification to the controllers implementation.	Creative Commons Attribution-ShareAlike 4.0 International License (CC BY-SA 4.0)
*RaspyControlLab-software.zip*	Folder with all software components to install in the Raspberry Pi OS from scratch.	Creative Commons Attribution-ShareAlike 4.0 International License (CC BY-SA 4.0)

## 5 Bill of materials

The complete Bill of Materials (BOM) is available in the open repository (https://doi.org/10.5281/zenodo.7500242). Some components, especially those for the tank system were made by a local manufacturer. Please, build these components with your local manufacturer or distributor with the dimensions specified in both the BOM and the 3D TinkerCAD model.

In the design, we used two power supplies for the motor pumps and the signal conditioning circuit. Nonetheless, one power supply can be included to reduce the costs with 12 V and 5 V support. By the same token, the Raspberry Pi 4 (4 GB) can be replaced by another of 2 GB since the RAM consumption of *RaspyControl Lab* does not exceed this value. Indeed as it is indicated in Section 8.5, the current RAM consumption for the experiment is 290 MB. The total implementation cost was USD 461, but with the previous modifications, the cost could be reduced to USD 420. In the BOM (l = length, h = height, w = width).

### 5.1 Tank system

**Table:** 

**Designator**	**Quantity**	**Cost per unit-USD**	**Total cost-USD**	**Source of materials**	**Material type**
12 V Brushless DC motor Pump	2	13.53	27.06	https://www.aliexpress.com/item/32472119699.html	Other
Ultrasonic sensor (US-016)	1	2.38	2.38	https://es.aliexpress.com/item/1005001593889019.html	Other
L298 driver	1	5.65	5.65	https://bit.ly/3NzYAYE	Other
Acrylic Tanks (h=51cm,w=20.8cm,thickness=4mm)	2	80	160	Local Manufacturer	Acrylic
Acrylic lids (dia = 20.8 cm, thickness = 4 mm)	2	10	20	Local Manufacturer	Acrylic
Acrylic Hinge (w=45mm)	2	4	8	Local Manufacturer	Acrylic
Plastic wiring duct with adhesive (w=1.5cm,h=1cm,l=1.5m)	1	4	4	Local Manufacturer	Plastic
Plastic hose (dia=1cm,l=2m)	1	10	10	Local Manufacturer	Plastic
Thread plastic ball valve $1/2^{\prime \prime }$ in	2	8.56	17.12	https://bit.ly/3a1HmWJ	Plastic

### 5.2 Signal conditioning circuit

**Table:** 

**Designator**	**Quantity**	**Cost per unit-USD**	**Total cost-USD**	**Source of materials**	**Material type**
ADS1115 (16-bit ADC)	1	14.95	14.95	https://www.adafruit.com/product/1085	Other
MCP6004 ($U_{1}$)	1	0.69	0.69	https://bit.ly/3a8bwaL	Semi-conductor
10 KΩ resistors (0.25 W) ($R_{1}$-$R_{4}$)	4	0.14	0.56	https://bit.ly/3y9fGan	Carbon film
1 KΩ resistor (0.25 W) ($R_{5}$)	1	0.14	0.14	https://bit.ly/3y9fGan	Carbon film

**Table:** 

**Designator**	**Quantity**	**Cost per unit-USD**	**Total cost-USD**	**Source of materials**	**Material type**
10 KΩ trimmer	1	2.04	2.04	https://bit.ly/3OPxjTe	Carbon film
Red led (3 mm) ($D_{1}$)	1	1.22	1.22	https://bit.ly/3AiNwMK	Other
Input capacitor (100 uF/25 V)($C_{1}$)	1	0.35	0.35	https://bit.ly/3OOMuMq	Electrolytic
3.3 V Zener diode ($D_{2}$)	1	1.257	1.257	https://bit.ly/3ezudGk	Semiconductor
14-pin IC socket	1	0.5	0.5	https://bit.ly/3OSBCx1	Plastic-metal
10-pin single row female header (pitch 2.54 mm)	1	0.75	0.75	https://bit.ly/3y7BFhR	Plastic-metal
3-pin JST XH connector set (pitch = 2.54 mm)-male connector and female cable	1	0.7	0.7	https://bit.ly/3uhiKA9	Plastic-cooper
4-pin JST XH connector set (pitch = 2.54 mm)-male connector and female cable	1	0.8	0.8	https://bit.ly/3OGuoNe	Plastic-cooper
Raspberry Pi GPIO Tall Header - 2x20	1	2.75	2.75	https://bit.ly/3An3Egl	Plastic-metal
Printed Circuit Board (PCB) for the conditioning circuit	1	16	16	http://schustercircuitos.com/ (Local manufacturer)	Polymer-copper

### 5.3 Camera support

**Table:** 

**Designator**	**Quantity**	**Cost per unit-USD**	**Total cost-USD**	**Source of materials**	**Material type**
$1/2^{\prime \prime }$ PVC pipe, l=1.5m	1	6	6	Local manufacturer	Plastic
$1/2^{\prime \prime }$ rounded elbow PVC coupling connector	1	3	3	Local manufacturer	Plastic
$1/2^{\prime \prime }$ T-shaped PVC coupling connector	1	3	3	Local manufacturer	Plastic
$1/2^{\prime \prime }$ PVC male threaded adapter with nut	1	3	3	Local manufacturer	Plastic

### 5.4 Raspberry Pi

**Table:** 

**Designator**	**Quantity**	**Cost per unit-USD**	**Total cost-USD**	**Source of materials**	**Material type**
Raspberry Pi 4 (4 GB)	1	55	55	https://www.adafruit.com/product/4296	Other
Raspberry Pi Camera Board v2 - 8 Megapixels	1	29.95	29.95	https://www.adafruit.com/product/3099	Other
Flex Cable for Raspberry Pi Camera or Display - 2 meters	1	5.95	5.95	https://www.adafruit.com/product/2144	Plastic-metal
Raspberry Pi 4 Power Adapter 5 V 4A Charger 20 W USB Type C	1	9.13	9.13	https://bit.ly/3I3fn5r	Other
Micro SD card (32 GB)	1	6.69	6.69	https://amzn.to/3KQefU3	Other

### 5.5 Power supply

**Table:** 

**Designator**	**Quantity**	**Cost per unit-USD**	**Total cost-USD**	**Source of materials**	**Material type**
Power Supply 1 (5 V)-Q-60B Four Output Switching Power Supply 60 W 5 V/12 V/-5 V/-12 V	1	11.2	11.2	https://bit.ly/3a0SDql	Other
Power Supply 2 (12 V)-3A DC adapter with connector	1	5.6	5.6	https://bit.ly/3uhg19H	Other

### 5.6 Components’ box

**Table:** 

**Designator**	**Quantity**	**Cost per unit-USD**	**Total cost-USD**	**Source of materials**	**Material type**
Acrylic components’ box (h=9.5cm,w=40cm,l=20cm) with top lid	1	30	30	Local manufacturer	Acrylic

## 6 Build instructions

In this section, we addressed the build instructions for the hardware and software. Firstly, we indicate the overall instructions. Secondly, we describe the particular instructions for each main component in hardware and software with different images that illustrate the procedure.

### 6.1 Overall instructions

In general terms, the instructions to build the hardware and software are summed up as follows: *For hardware:*

1. Build the acrylic tanks and components’ box with the dimensions and holes depicted in the TinkerCAD 3D model taking into account the 1:10 scale.
2. Put and stick the motor pumps in the acrylic tanks. Attach the hoses to the motor pumps and pass them between the tanks, using the holes in the top part of each tank.
3. Put and stick the ultrasonic sensor (US-016) in the top lid of the main control tank (T1).
4. Stick the wiring duct in each tank to organize the wires of each motor pump.
5. Solder the components of the PCB. Follow the Proteus VSM schematic. For the MCP6004 use a 14-pin IC socket. For the ADS1115 employ a 10-pin single row female header connector (1*10-pitch = 2.54 mm).
6. Put the Raspberry Pi, signal conditioning circuit, L298 driver, and power supplies in the components’ box. Attach them to the box employing M3 or M2.5 screws with their nuts.
7. Wire each other the previous components. Please, pay special attention to the wiring connections of the power supplies (12 V, 5 V) and the common ground between the hardware components.
8. Turn on the power supplies. If necessary, check the voltages of these, the L298 driver, and the signal conditioning circuit with a multimeter. Before connecting the ADC, check that the output of the signal conditioning circuit does not exceed 3.3 V.

*For software:*

1. Download and save the Raspberry Pi OS image available in the repository of the project on a micro SD card (32 GB). For this task, employ the software Raspberry Pi imager.
2. Plug in the micro SD to the Raspberry Pi 4.
3. Attach the 5 V power supply of the Raspberry Pi 4. Wait for the Raspberry Pi OS starts.
4. By default the IP of the Raspberry Pi is assigned by DHCP. If you need to change this IP, use the software advanced IP scanner to detect the current IP. Install a VNC client and access to this IP with the following credentials: (User: **pi**, Password: **remotelab**). Change the IP directly in the Raspberry Pi OS according to your needs.
5. Search and modify the **line 191** with your assigned IP in the following file:/var/www/FlaskApp/templates/indexcode.html. Use nano, Geany, or another editor. Next, save this file and type from a terminal in the Raspberry Pi OS the command **sudo service apache2 restart**.
6. Type the IP of your Raspberry Pi from your browser or access the domain name (http://raspberrypi). Check the real-time video of the experiment. Utilize the different Python scripts available in the repository to test the automatic control experiment.

### 6.2 Hardware installation instructions

#### 6.2.1 Installing Acrylic tanks, motor pumps, and ultrasonic sensor

**Fig. 11:**
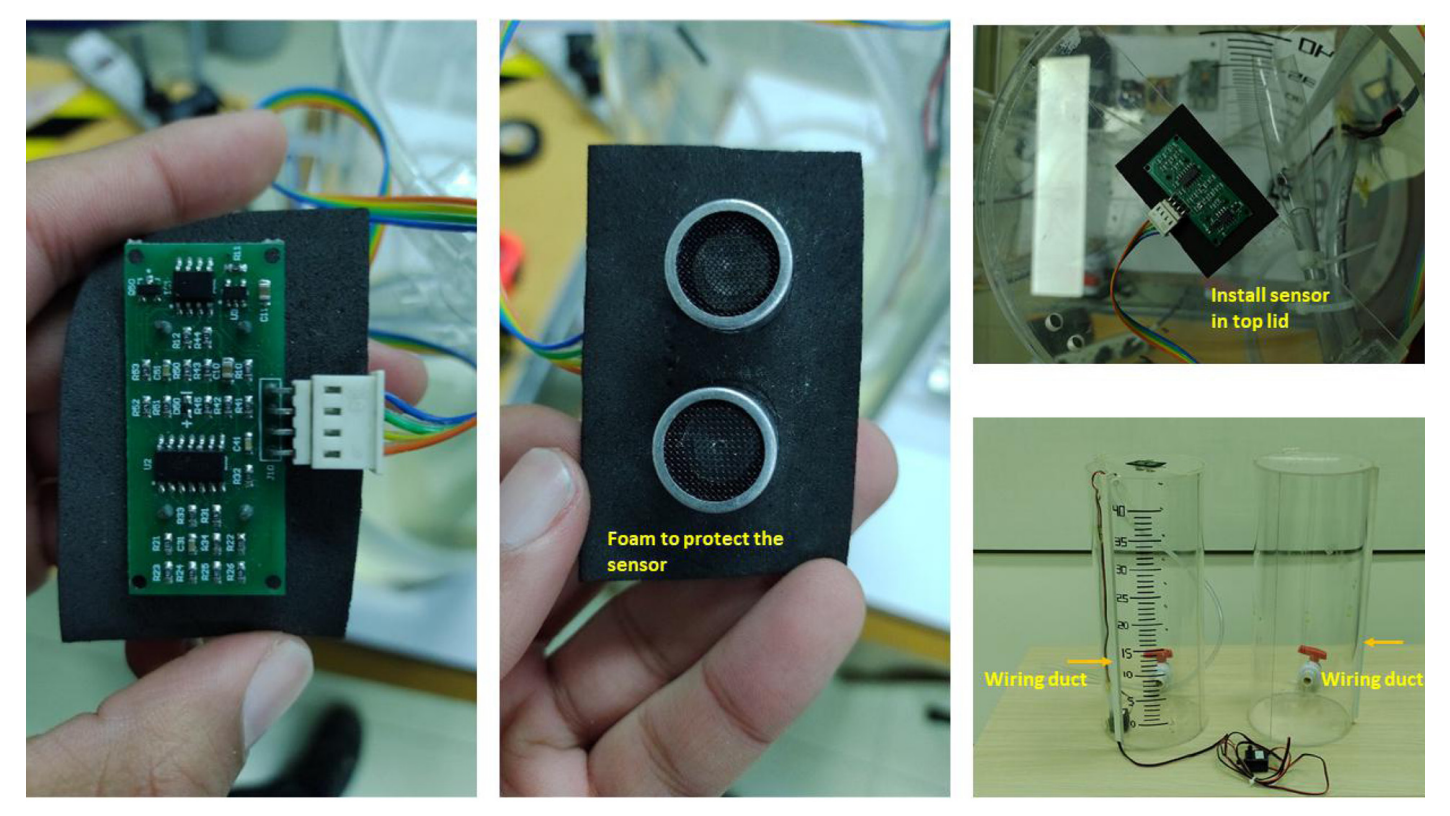
Process of attaching the ultrasonic sensor to the top acrylic lid.

1. Create the tanks with the dimensions specified in the TinkerCAD model. Stick the plastic ball valves in the indicated holes at the bottom part of each tank. The top lids for both tanks employ acrylic hinges. Stick them with an adhesive, e.g., Loctite Super Bonder. Stick the ultrasonic sensor (US-16) in the top lid of the tank (T1) with the holes in this. Use a foam piece of the sensor’s size to protect or replace it easily if necessary. Stick the adhesive PVC wiring duct in each tank with the dimensions (h=10mm,w=15mm,l=48cm) to organize the wires of both each motor pump and the ultrasonic sensor. See Fig. (11) and Fig. (12).

**Fig. 12:**
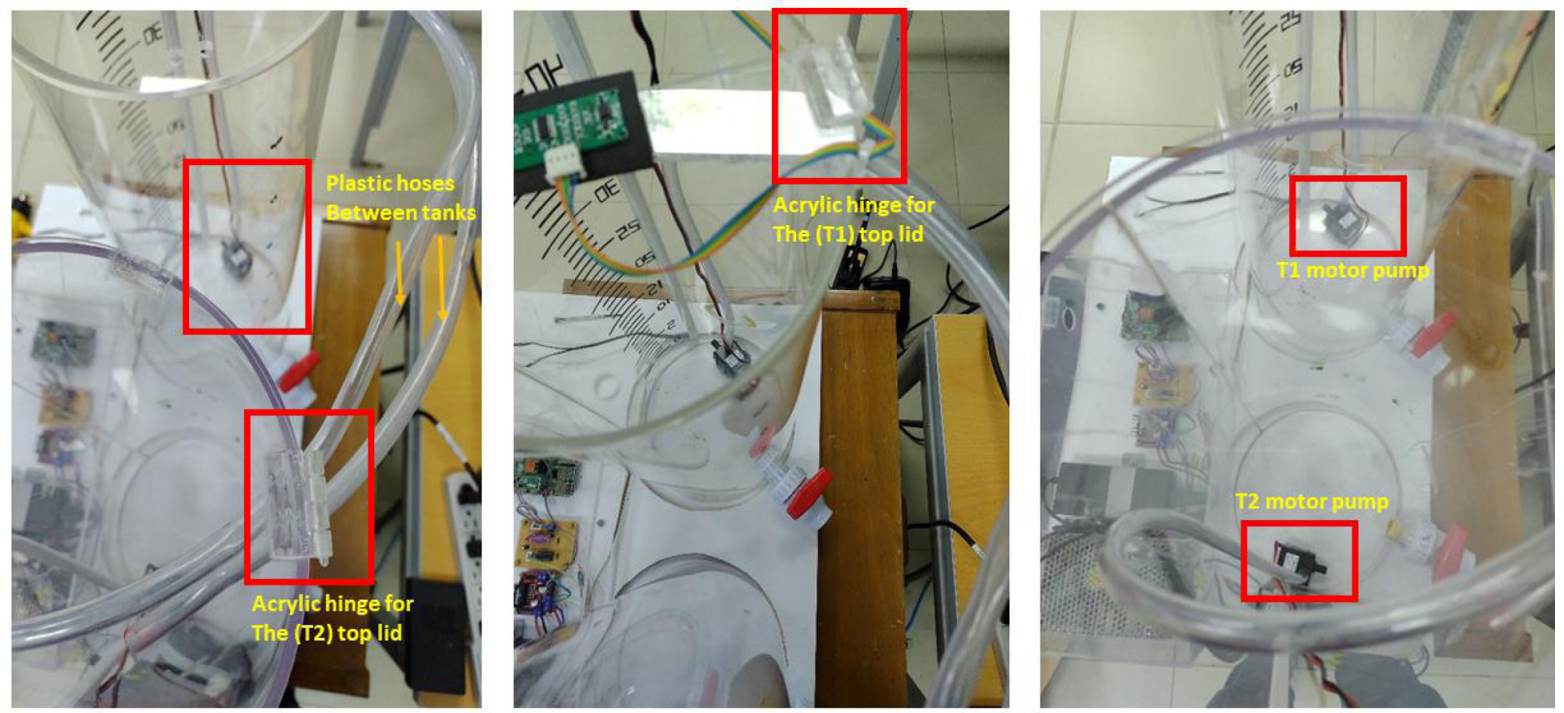
Procedure to install motor pumps, plastic hoses, and acrylic hinges.
2. Install the motor pumps in the bottom part of the acrylic tanks. Connect the plastic hoses (diameter=1cm) to the motor pumps with an adequate length. Pass the hoses between the tanks, using the holes in the top part of each tank. To reduce the liquid turbulence in the tank (T1) that could affect the measurement of the ultrasonic sensor, cut a small piece of transparent plastic acetate, and install it in the final portion of the plastic hose that deposits liquid in the tank T1. Join this plastic piece to the hose with a nylon cable tie. See Fig. (12), and Fig. (13). Similarly, with help of a measuring tape, draw or stick the level ticks in centimeters for (T1), taking into account that the minimum level for the motor pump of (T1) is**4.5 cm over the bottom base of the tank** as mentioned. (See Fig. (14))

**Fig. 13:**
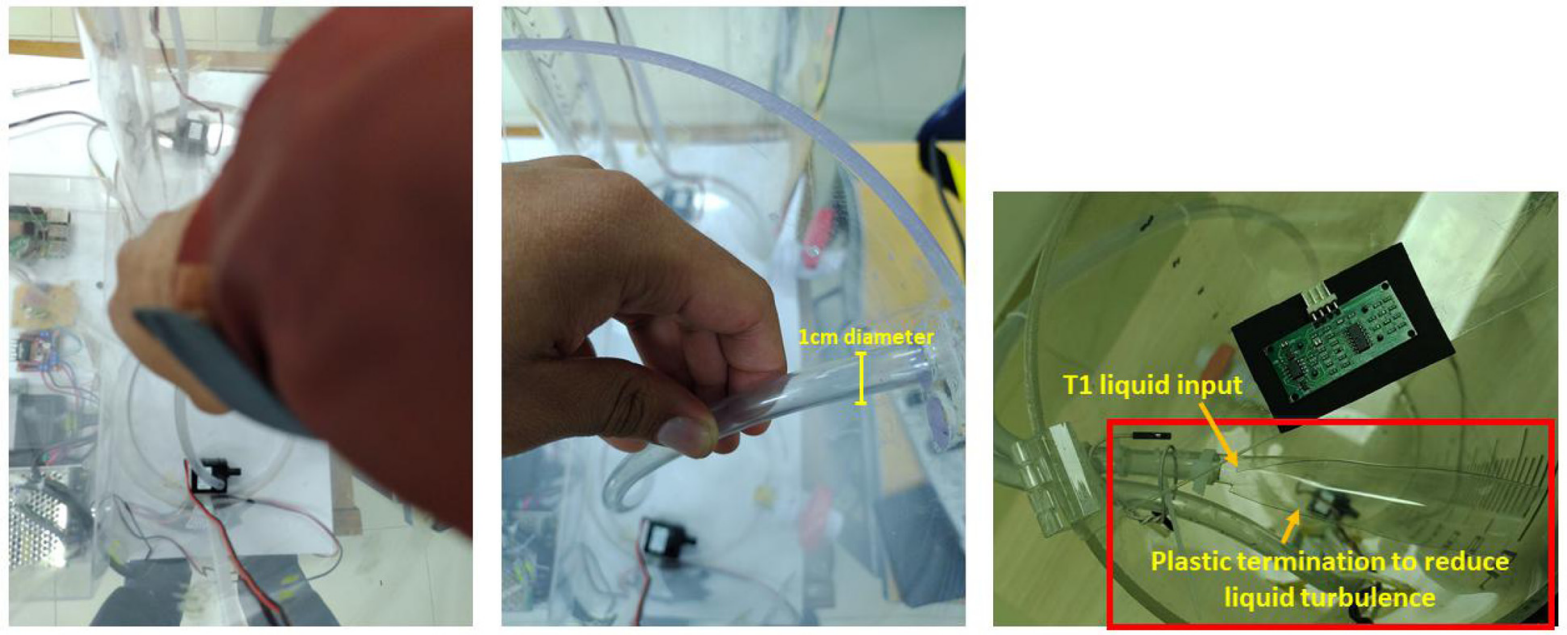
Procedure to install plastic hoses, and transparent plastic acetate termination to reduce turbulence in tank (T1).

**Fig. 14:**
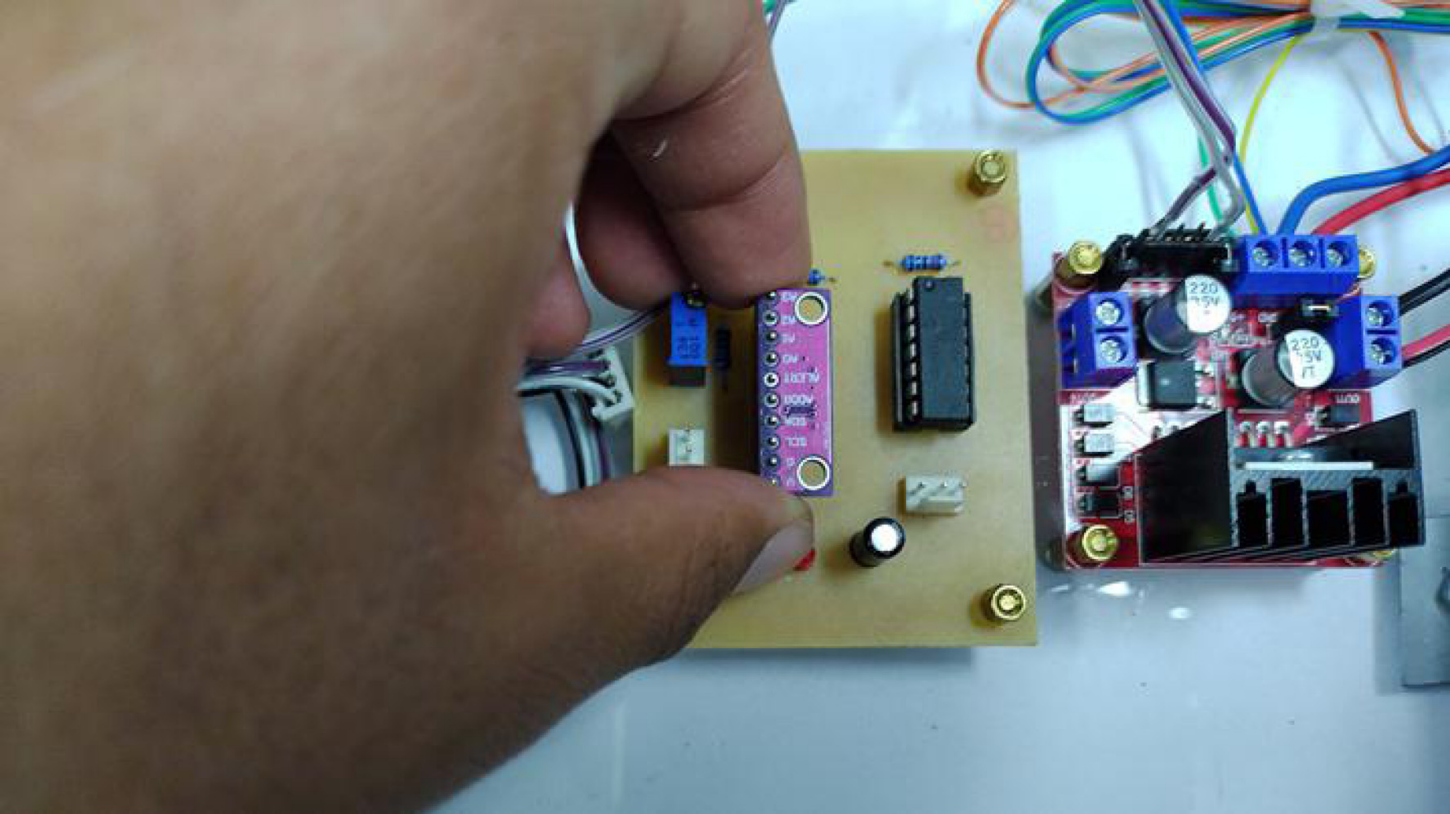
Signal conditioning assembling procedure for ADS1115.

#### 6.2.2 Assembling the signal conditioning circuit

Solder the components of the signal conditioning circuit according to the schematic and PCB designs provided in the repository in Proteus 8.9 VSM. The MCP6004 and $\mathrm{ADS}1115_{\mathrm{IN}}$ connections employ JST XH connectors (pitch = 2.54 mm) of 3-pin and 4-pin, respectively. Similarly, for the Operational Amplifier (OP-AMP) MCP6004, use a 14-pin IC socket. For the ADS1115, employ a 10-pin female header connector (1*10-pitch = 2.54 mm). Attach the ADC (ADS1115) and the MCP6004 to the PCB of the signal conditioning circuit. Besides, notice that we utilized precision resistors in the PCB (1% tolerance).

#### 6.2.3 Installing the components’ box

**Fig. 15:**
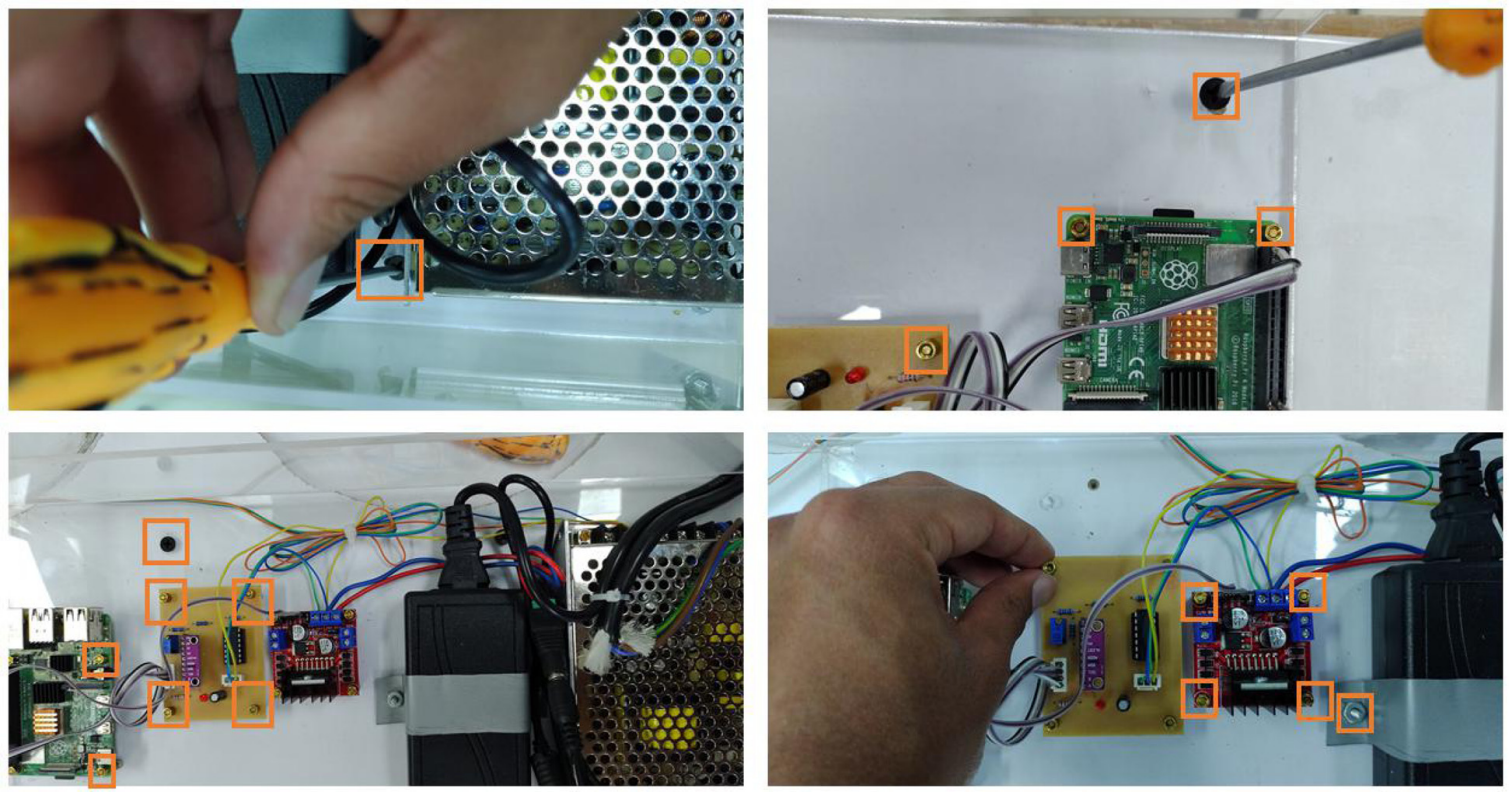
Procedure to attach the circuits to the components’ box through M2.5 or M3 screws and their nuts.

Open the holes for the M3 screws of the L298 driver, and the signal conditioning circuit in the bottom part of the components’ box. Also, open four holes for M3 screws to attach the acrylic box to a wood or acrylic base. Regarding the Raspberry Pi, employ M2.5 screws. To connect the camera support, open a 0.5-inch hole on the front of the components box. Locate the components in the box and take as reference the TinkerCAD 3D model. To assure the components, use either M3 or M2.5 nuts and screws as the orange squares in Fig. (15) depict according to the component.

#### 6.2.4 Making wiring connections

**Fig. 16:**
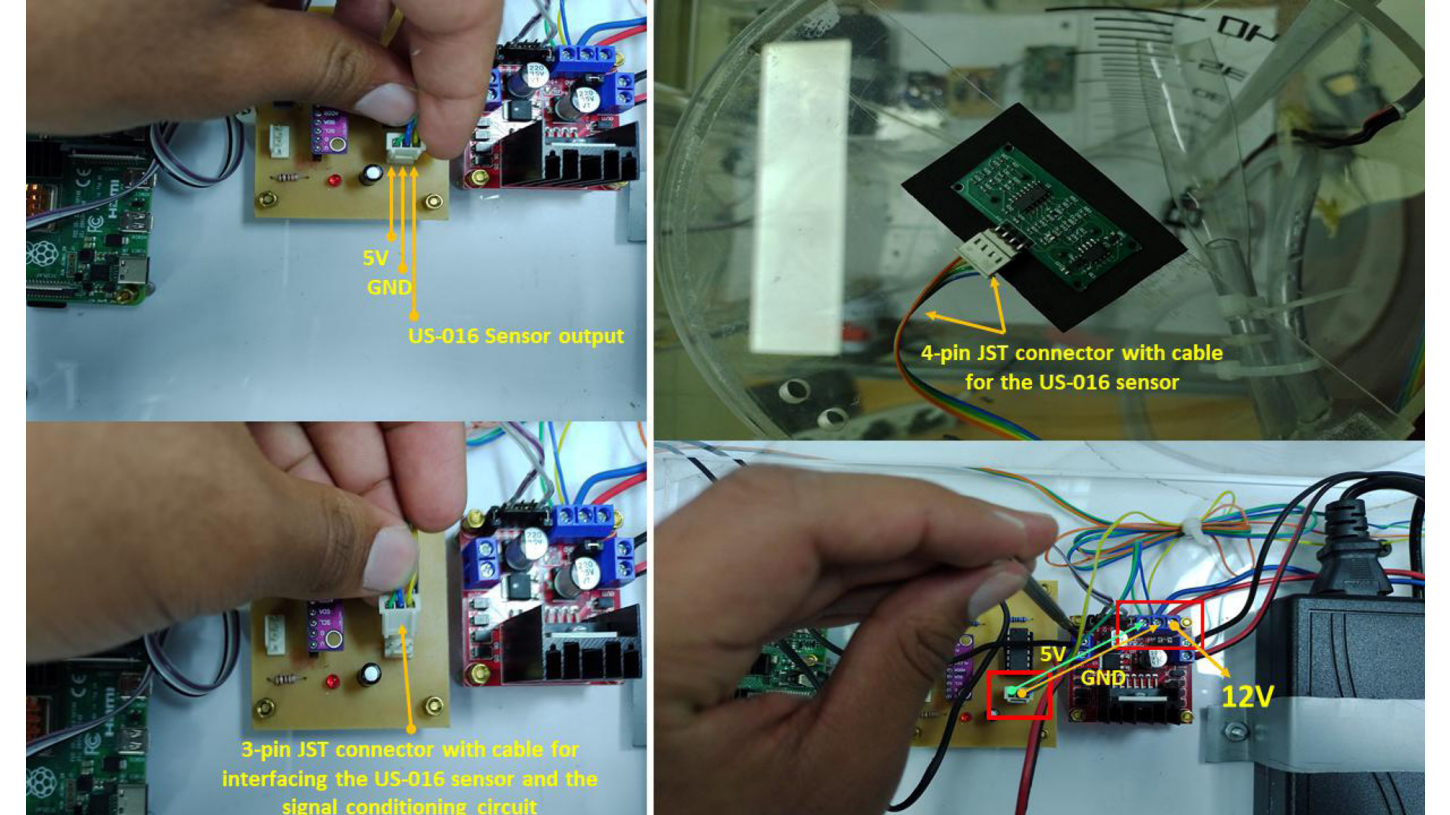
Wiring connections for the ultrasonic sensor and signal conditioning circuit.

**Fig. 17:**
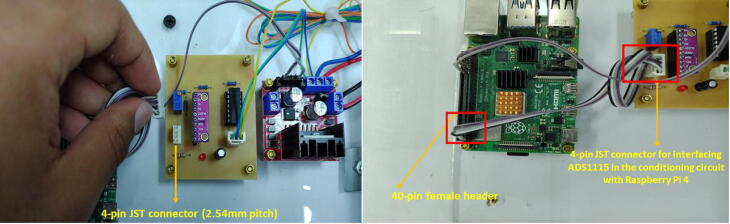
Wiring connections for interfacing ADS1115 with the Raspberry Pi 4.
• Make a cable with a 4-pin JST XH connector (2.54 mm pitch) for interfacing ADS1115 with the Raspberry Pi 4. Solder the wires of this cable to the respective pins in the Raspberry Pi GPIO Tall Header – 2 × 20. Follow the schematic of the signal conditioning circuit, remaining that the ADS1115 is interfaced to the $I^{2}C$ protocol in the Raspberry Pi. Also, connect the 3.3 V power supply of the Raspberry Pi to the respective pin of this cable.
• Attach the motor pump wires to each terminal block in the L298 driver. Make a cable to interface the GPIOs for the PWM control of the motor pumps with the L298 driver. Follow the connections described in Fig. (6), remaining that GPIO12 controls the motor pump for T1 (control plant), while GPIO13 for T2 (reservoir tank). The connections of the motor pumps should have the polarity indicated in Fig. (18).

For the wiring connections, please follow these instructions:

• Make a cable with a 4-pin JST XH connector (2.54 mm pitch) for the ultrasonic sensor with l=1.5m. This cable will be attached to the sensor in the top lid of the tank (T1). Make a second cable with a 3-pin JST XH connector (2.54 mm pitch) for the ultrasonic sensor with a l=30cm. This last connector will be attached to the PCB of the signal conditioning circuit. Both cables only have a JST XH connector on one side. To make the connections of the 5 V and GND for the sensor for both the (T1) top lid and the signal conditioning circuit, we employed the same terminal block of the L298 driver. Besides, we attached in this terminal block, the power supplies (12 V, 5 V) as Fig. (6) and Fig. (16) show. Pay attention to these power supply connections. Join the $V_{\mathrm{out}}$ of the ultrasonic sensor with the respective pin in the 3-pin JST XH connector of the conditioning circuit (See Fig. (17)).

**Fig. 18:**
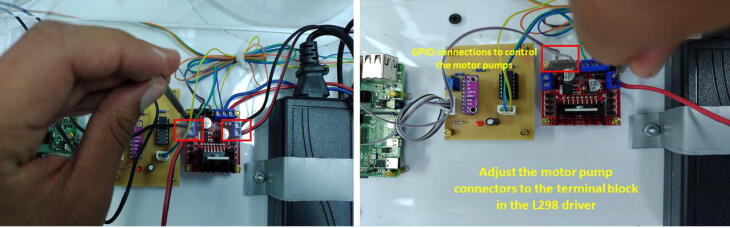
Wiring connections for interfacing GPIO PWM control pins and L298 driver.

#### 6.2.5 Build camera support

**Fig. 19:**
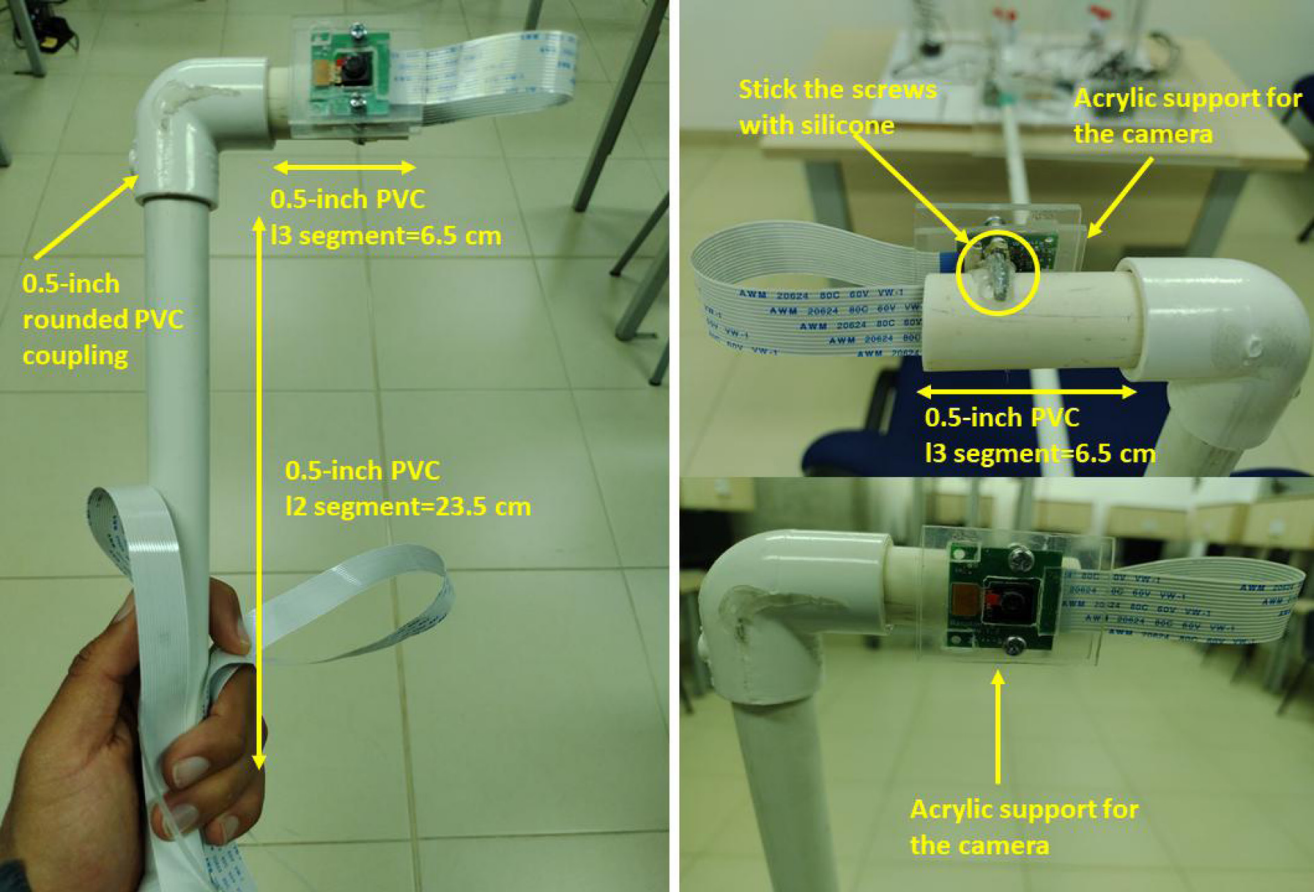
Instructions to assemble the camera support. Part I.
• Pass the 2 meters flex cable of the Raspberry Pi camera through the PVC pipes $l_{2}$ and $l_{3}$.
• To connect the PVC pipe segments ($l_{1}$) and ($l_{2}$) use a 0.5-inch T-shaped PVC coupling connector. First pass the flex cable through the $l_{1}$ segment, next join the segments. (See Fig. (20))

The camera support was created with PVC pipes and couplings. The instructions to assemble it are the following:

• Cut three segments of 0.5-inch PVC pipe with $l_{1}=78\;\text{cm},l_{2}=23.5\;\text{cm},l_{3}=6.5\;\text{cm}$, respectively. Specifically, to support the camera, join the segments $l_{2},l_{3}$ with a 0.5-inch rounded elbow coupling pipe. Stick the screws of the acrylic camera support to the PVC ($l_{3}$) segment as Fig. (19) illustrates.

**Fig. 20:**
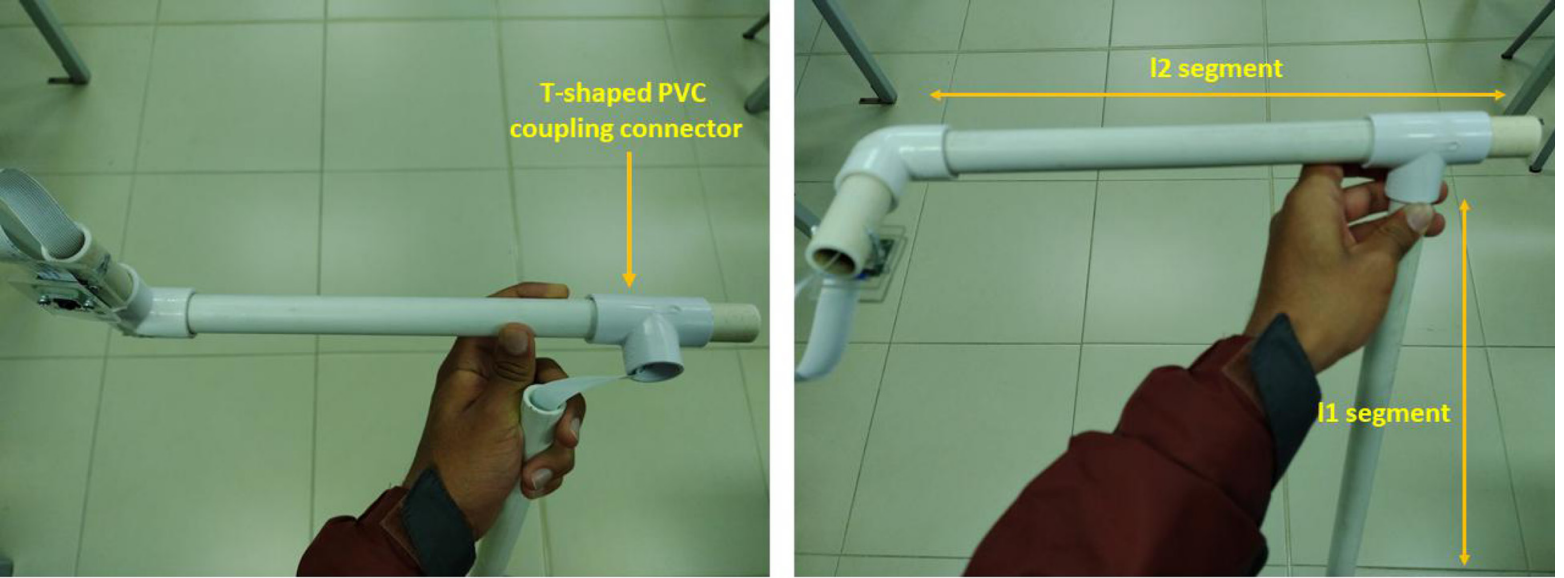
Instructions to assemble the camera support. Part II.
• To connect the PVC pipe of the camera, open a 0.5-inch hole in the front side of the components’ box. Attach a 0.5-inch PVC threaded male adapter with a nut to support the PVC pipe $l_{1}$. Pay attention to this part because the weight of the camera support and the PVC pipe could tear or break the acrylic components’ box. To avoid this issue, put the PVC support on a desk, chair, etc. (See Fig. (21))

**Fig. 21:**
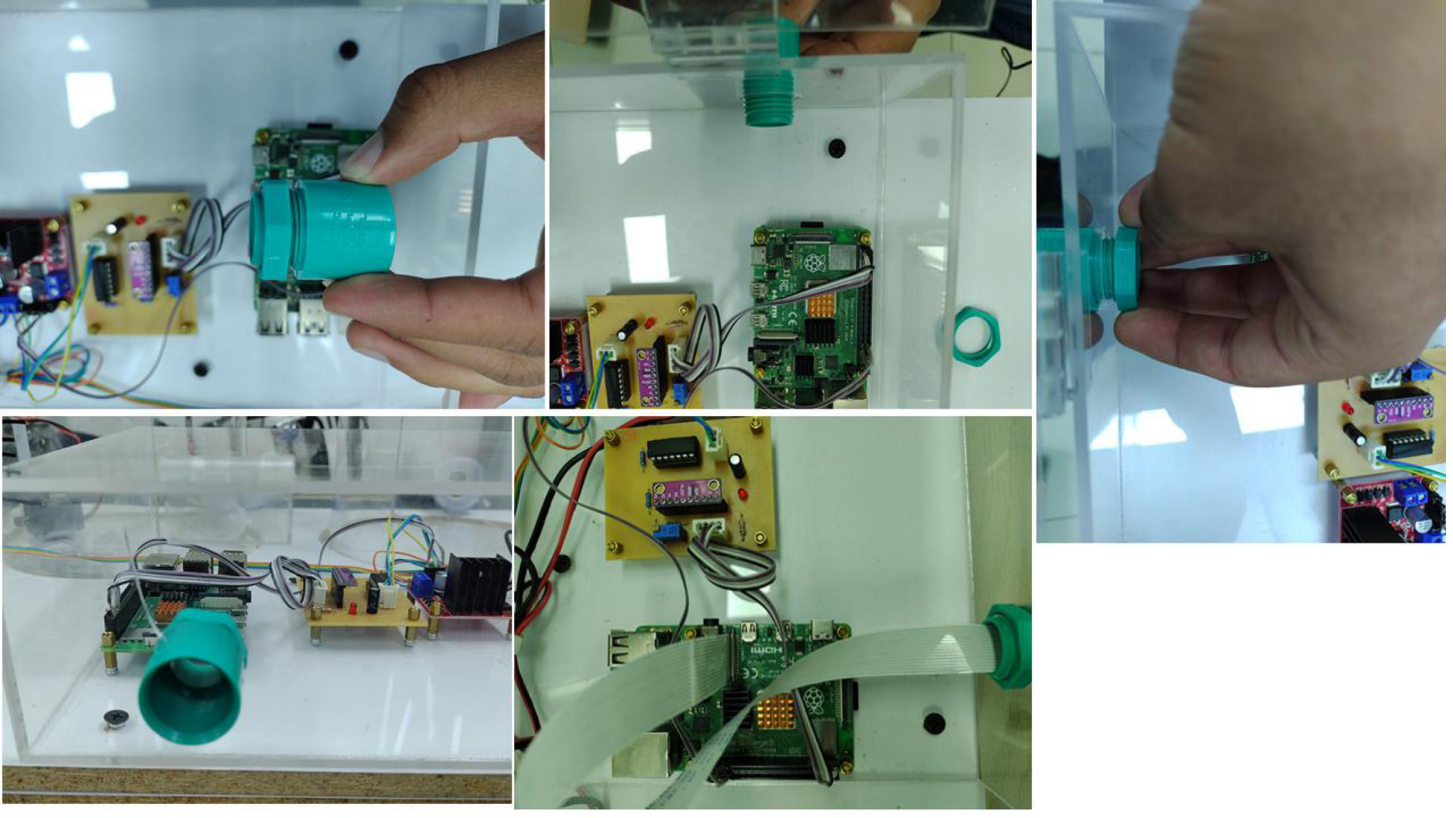
Instructions to assemble the camera support. Part III.
• Finally, connect the flex cable to the Raspberry Pi camera connector.

### 6.3 Software installation instructions

**Fig. 23:**
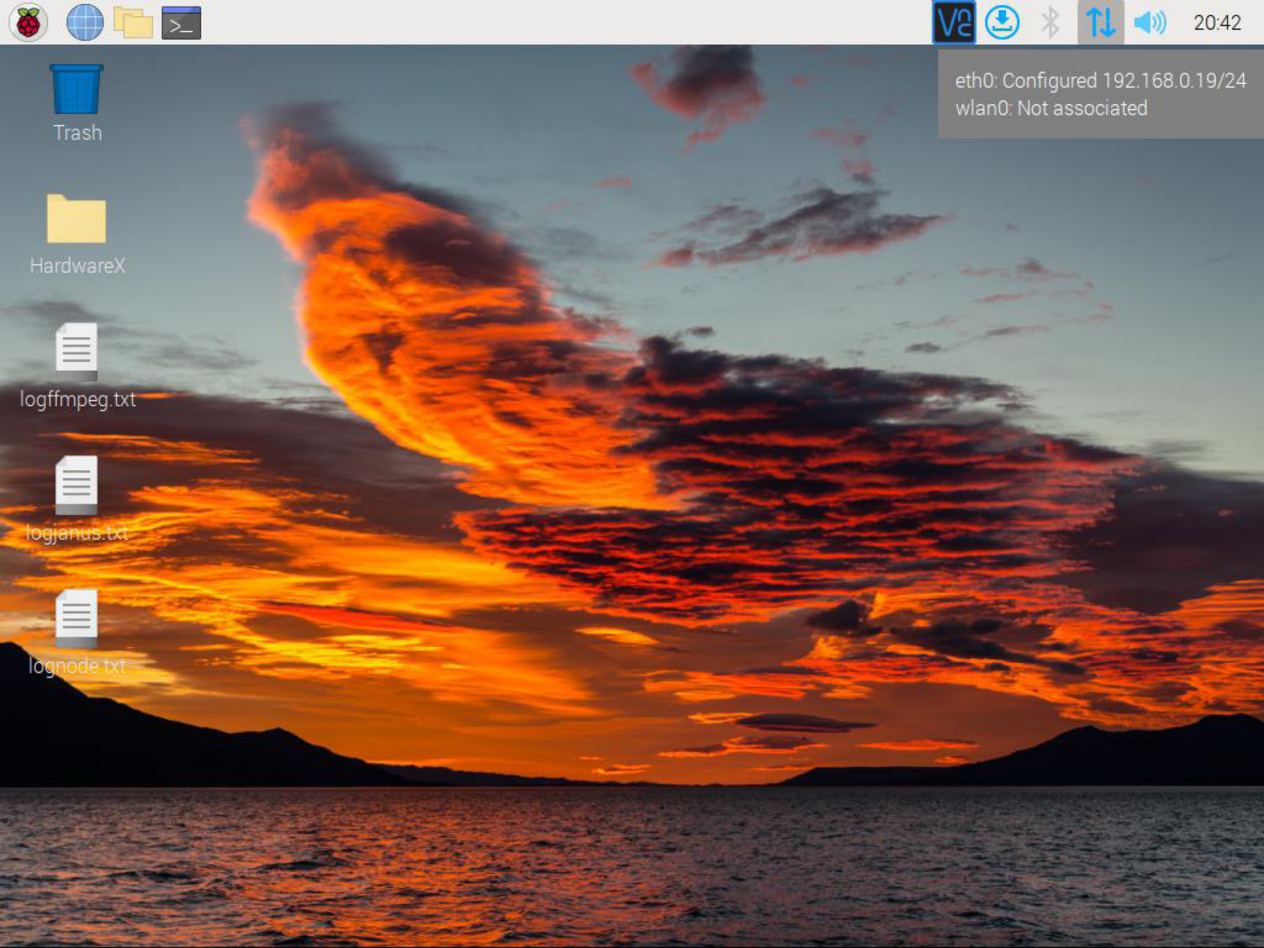
Example of IP assigned to the Raspberry Pi through Ethernet connection.

In this section, we entailed the software instructions for educators, users, or developers. (See Fig. (23)).

#### 6.3.1 Instructions for educators or users

1. Download the Raspberry Pi OS image from the repository of the project. Uncompress it in a folder destination. To save this image on the micro SD card, download and install the software Raspberry Pi Imager available at  https://www.raspberrypi.com/software/.
2. Once the software is installed, open it, and click on the button “choose OS”. Here, go to the last option in the menu ”use custom”. Then, search for the image downloaded in step 1, and click on open.
3. To select the micro SD card, click on the button “choose storage”. Next, click on the button “Write”. This process will write the image on your micro SD card. Take into account that your micro SD card should have a minimum size of 32 GB. If there is a size problem with the SD card, use another of 64 GB. Wait for the process to finish. Fig. (22) describes this overall procedure.

**Fig. 22:**
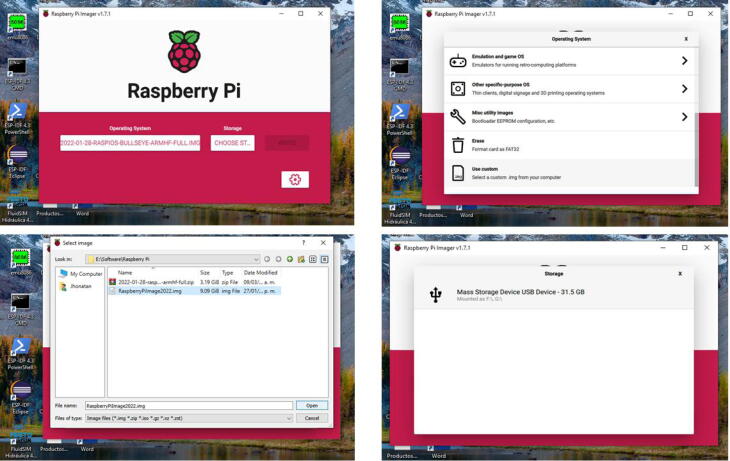
Procedure to write the Raspberry Pi Os image in the micro SD card.
4. Insert your micro SD card with the written image in the slot of the Raspberry Pi. Next, plug in the 5 V power supply thereof. The Raspberry Pi OS image contains all software components in order to start up the *RaspyControl Lab*.
5. To connect the Raspberry Pi to the network, you must have an available router, server, or access point. By default, the Raspberry Pi takes an IP through DHCP protocol, that is, whatever of the previous devices must assign an IP automatically to your Raspberry Pi. However, *RaspyControl Lab* needs an initial setup configuration, taking as a parameter this IP. To connect your Raspberry Pi to the network, you have two options.
  The first one is to connect directly your Raspberry Pi to a TV, monitor or similar through the micro HDMI connector. Then, you can either select the SSID of your network to connect the Raspberry Pi through WiFi or use the Ethernet connector. In any case, please pay attention to the IP that is assigned to the Raspberry Pi.
  The second one requires a wired connection (Ethernet connection) to use a VNC viewer to access to the Raspberry Pi OS. In this case, a VNC server is installed and ready to use in the OS image. If you choose this option, please go to the following link to download the VNC viewer:  https://www.realvnc.com/en/connect/download/viewer/. For the VNC viewer, the credentials in the Raspberry Pi are (User: **pi**, Password: **remotelab**). To detect the current IP of the Raspberry Pi, use the software advanced IP scanner which is available at  https://www.advanced-ip-scanner.com/.
6. With the previous IP configured in your Raspberry Pi, go to the location **/var/www/FlaskApp/templates** and edit the file **indexcode.html**, employing Geany or a text editor. Go to line 191 and change the IP. Then save the file. For this process, see Fig. (24). The file indexcode.html contains the webpage for the users to interact with the experiment.

**Fig. 24:**
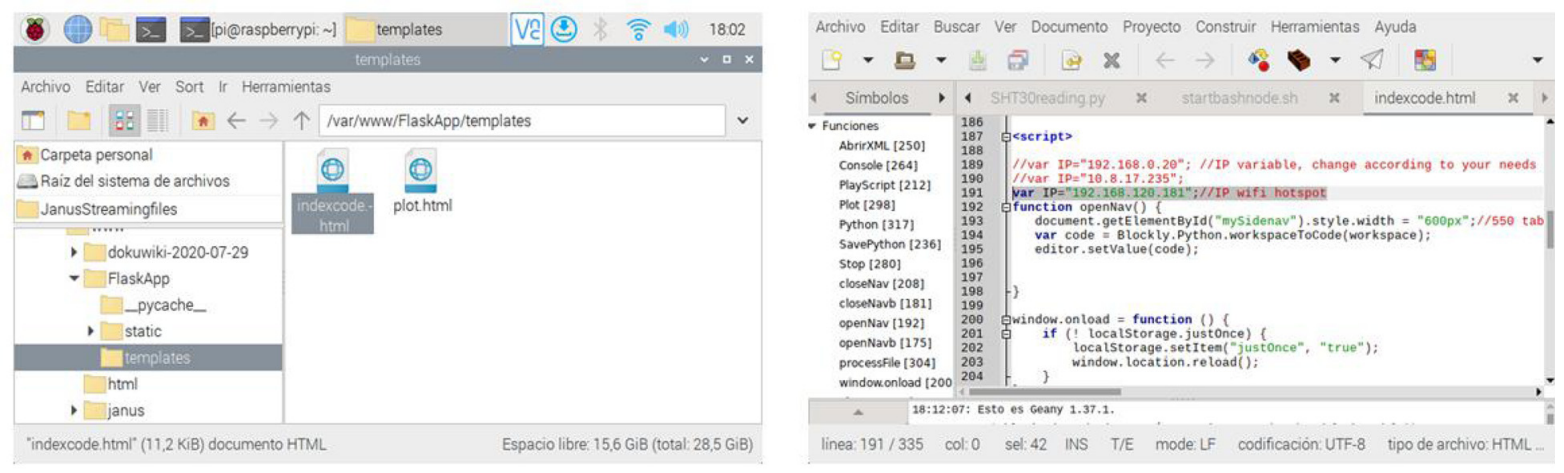
Example of IP configuration in the file indexcode.html.
7. Go to a terminal and write the command to restart the Apache server: **sudo service apache2 restart**. At this point, the setup configuration is ready.

#### 6.3.2 Instructions for developers

For developers that want to install all software components from scratch using a custom Raspberry Pi OS image, please see the instructions of the GitHub repository of the project available at  https://github.com/Uniminutoarduino/RaspyControlLab.

## 7 Operation instructions

To access and experiment with *RaspyControl Lab*, follow these instructions:

1. Fill with liquid the tank (T2). Turn on the power supplies (5 V, 12 V).
2. Plug in the DC adapter of the Raspberry Pi. Wait for the Raspberry Pi OS starts.
3. Open a web browser (Google Chrome or Mozilla Firefox) and type the IP of your Raspberry Pi. Also, you can access *RaspyControl Lab* typing the hostname  http://raspberrypi.
4. Check the output voltage of the OP-AMP MCP6004, which is interfaced to the channel A0 of the ADS1115. To the minimum level of T1, the output voltage must be approximately 0 V. Adjust this voltage with the trimmer resistor in the circuit. To achieve this minimum level, use the Python script “fill_tank.py” to turn on the motor pump of the tank (T1) and reach the minimum level of liquid in this. You can pass liquid between tanks enabling the correct GPIO output. GPIO12 to control the main tank (T1) and GPIO13 for the reservoir tank (T2).
5. To interact with the experiment, you can use the scripts for the different controllers that we have designed. These are available in the main repository of the project. See Section 4. To run any script, click on the run button on the webpage.
6. To see the Python console, click on the button ”view python console”. You must create a print statement with the text or variable to show in the console. Once you have tested the script in the console, you could click on the stop button to end the script execution.
7. To plot real-time data, we have created a small python library known as **plotter**. You can import this library in your Python script as follows:

**Table:** 

import plotter as plot # Library to plot data
#You can use any of the following functions as you desire:
plot.1m(str(sensor)) #Plot a sample of one sensor, controller, etc.
plot.2m(str(sensor),str(sensor2)) #Plot two samples of sensors, controller, etc.
#Plot three samples of sensors, controller, etc.
plot.3m(str(sensor),str(sensor2),str(sensor3))

The library contains three methods to plot up to three samples of sensors, controllers, etc. The arguments of these methods are data in form of a “string”. Therefore, we employed the method “str” to transform any numeric variable into a string. If you experiment any trouble with the plotting option, please go to the Section 7.2 for troubleshooting.

If you have a doubt about the previous process, please consult the following tutorials created for the project:

**Table:** 

**Video name**	**Description**	**Link**
*Rasperry Pi Imager*	A video about how to configure and use the Raspberry Pi imager to write the Raspberry Pi OS image of the project.	https://youtu.be/YXvWhFYZryM
*Access RaspyControlLab*	A video that shows how to access *RaspyControl Lab*.	https://youtu.be/nNIj8CU-z-w
*Change IP*	A video that depicts how to configure the IP in order to access and utilize the remote experiment.	https://youtu.be/RzXFiwRuPsQ
*PI Controller*	A video that depicts an example of PI controller employing *RaspyControl Lab*.	https://youtu.be/mceNmW32gA0
*PI controller with plotter*	A video that illustrates how to use the plotter of *RaspyControl Lab*.	https://youtu.be/ifcukqjerqE
*Complete test (PI controller)*	A video that illustrates a test for the PI controller in the laboratory setting.	https://youtu.be/Vvyo_BSJTMU

### 7.1 Internet connection alternatives for *RaspyControl Lab*

As mentioned, we tested *RaspyControl Lab* in a LAN. To provide access to the experiment on the Internet, we suggest the following:

1. The simplest method is to get a public IP for your Raspberry. In this case, change the IP of the Raspberry according to the previous steps in the sections on instructions for educators and developers. Also, take in mind that the hostname of the experiment is **raspberrypi**.
2. If you already count with an application server and you desire to incorporate RaspyControl Lab into it, please, redirect the IP of RaspyControl Lab to be accessed by it. This can be done using *rewrite* or *redirect* rules in the Apache server o Nginx server, possibly installed on your application server. However, take in mind the IP assignation in the sections on instructions for educators and developers. Similarly, if you plan to use a domain or subdomain, you need to use redirect rules in the Apache server o Nginx server to the IP of the Raspberry Pi. Some references to do that can be consulted in [34], [35], [36], [37].
3. The same system used in *RaspyControl Lab* was implemented in *RaspyLab*[21]. In this case, we deployed the remote laboratory in a Virtual Private Server (VPS) with web access through an internet connection to the students. Please, check the previous reference for further information.
4. To build a server with Raspberry Pi to provide an Internet connection to the experiment, please, follow the steps of the GitHub repository of this project. If another server is built on a Linux machine, e.g., on Ubuntu, install each component used in the project, for example, the Janus WebRTC server, etc., according to the instructions provided on the website of each developer for this Linux distribution.

### 7.2 Possible issues and troubleshooting

If you have any doubt about the software component of *RaspyControl Lab*, consult the GitHub repository  https://github.com/Uniminutoarduino/RaspyControlLab and follow the instructions. In some cases, if you configure the IP of the Raspberry Pi from the wireless and wired network settings in the Raspberry Pi OS, the IP could fail. In this case, change the IP of your Raspberry PI again, following the procedure in the video “Change IP” available above. At last, reboot your Raspberry Pi. Besides, be aware of the version of the browsers Chrome and Firefox to support the standard WebRTC. Please, see this webpage to know the minimum version for those:  https://caniuse.com/?search=webrtc.

Another possible issue is regarding the plotting option. We used a *redis* database to save the values of the data sent from the RL. If the plotting option fails, check both the status of the *redis* server and the node.js server. In this case, follow these steps:

• Close the plot window in your web browser. Always that you use this feature, stop sampling with the stop button on the webpage.
• Open a terminal in the Raspberry Pi OS.
• Write the command **ps aux** ∣ **grep node** to identify the node.js applications running on the Raspberry Pi.
• Kill the node.js processes using the command **sudo kill** −**9 (process number)**. Delete all processes listed from the previous command that are linked to the folder HardwareX on the desktop of the Raspberry Pi OS.
• Reboot your Raspberry Pi. This would fix the problem with the plotting option.

Similarly, if you plan to use a touchscreen monitor on the MIPI DSI display port of the Raspberry Pi, the VNCViewer could show a black screen. In this case, go to step 17 in the software repository of the project to solve this problem. If there is a problem with the ADC (ADS1115), please, check the voltage (3.3 V), the pins for the protocol $I^{2}C$, and their setting in the Raspberry Pi OS.

Finally, as an observation, the video can present some delays of a few milliseconds due to the LAN or Internet connections. This is a proper issue with the Janus WebRTC server and the clients’ video connections. In any case, this problem is solved automatically with the stabilization of the video frames over time. If the problem continues, just reload the webpage.

## 8 Validation and characterization

The validation process of *RaspyControl Lab* was made by performing the different steps in automatic control such as identification of the control plant, simulation of the identified model, controller design, and debugging. These stages allowed us to test both the tank control system and the user interface in Python language. Furthermore, the designed Simulink models and the SISO tool sessions from MATLAB R2018b employed in the stage of validation and characterization are available in the open repository of the project. In accompaniment with these models, datasets for the different experiments to contrast the theoretical and empirical data can be found in Section 4.

### 8.1 Plant and conditioning circuit identification

**Fig. 25:**
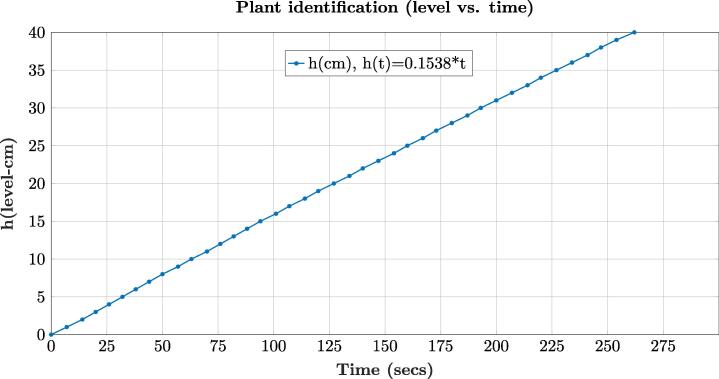
Plot of the level (h(cm)) vs. time. for the tank (T1).

**Fig. 26:**
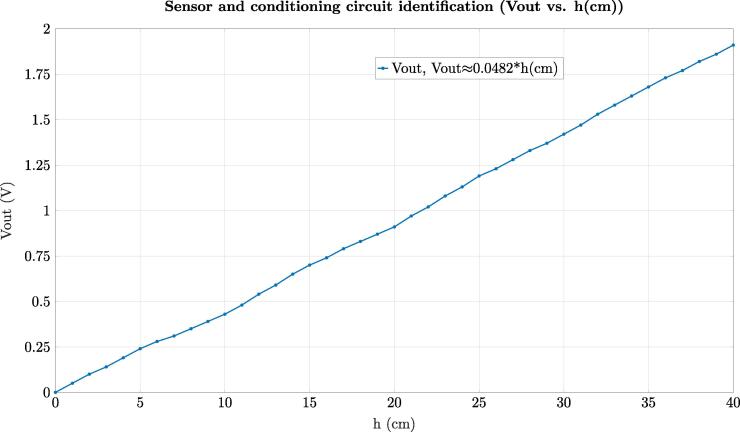
Plot of $V_{\mathrm{out}}$ for the conditioning circuit and sensor vs. level (h(cm)) of tank (T1).

One of the most important steps in automatic control is the identification of the control plant, that is, the mathematical behavior of the control tank system presented in Fig. (3). To identify this behavior, we conducted three experiments in which the tank (T1) was filled from the minimum level (0 cm) to (40 cm) that is the maximum level allowed in the control plant. After, the average of the data for the experiments concerning level (cm) vs. time (secs), and $V_{\mathrm{out}}$ of the conditioning circuit vs. level (cm) were plotted such as Fig. 25, Fig. 26 illustrate.

Initially, the mathematical expression that rules the behavior of the level of the tank (T1) is given by the following equation:

(1) $$Q(t)=A*\frac{\mathrm{dh}(t)}{d(t)}$$

where A is the tank area, h(t) is the level in function of the time, and Q(t) is the flow of liquid that enters the tank (T1) from the motor pump of the reservoir tank (T2). It is important to mention that the flow (*Q*) is the input of the control system whereas the level (*h*) is the output. The previous is because we need to find out the Laplace domain expression for the transfer function G(s) of the control system. Moreover, the internal diameter of the tank (T1) is 19.6 cm which yields to $A_{\left(T1\right)}=301.7{\mathrm{cm}}^{2}$. To identify the maximum flow *Q* in $\frac{{\mathrm{cm}}^{3}}{\mathrm{secs}}$ of the motor pump, we performed three experiments in which a level of h=20cm was reached in the tank (T1) with an applied voltage of 12 V, and the respective time was registered for each experiment. After, the values of volume in ${\mathrm{cm}}^{3}$ for the tank (T1) with their time were averaged. Then, the identified flow under these conditions was $Q(t)=47.77\frac{{\mathrm{cm}}^{3}}{\sec}$. A similar result can be achieved multiplying the slope of the expression presented in Fig. (25), $\frac{\mathrm{dh}(t)}{\mathrm{dt}}=0.1538$ with the area of the tank $A=301.7{\mathrm{cm}}^{2}$. Therefore, in this case, $Q(t)=46.4\frac{{\mathrm{cm}}^{3}}{\sec}$.

With the data of the experiments for the level *h* vs. time (secs) and the $V_{\mathrm{out}}$ vs. time (secs) of the conditioning circuit, it was identified the expressions indicated in the Fig. 25, Fig. 26 using linear regression due to the behavior exhibit by these variables in the trials. This procedure allowed us to identify the transfer function of the tank control system G(s) and the gain of the conditioning circuit H(s) in Laplace domain (s) as follows (see Fig. 25, Fig. 26), and the expression (1)):

(2) $$G(s)=\frac{Y(s)}{Q(s)}=\frac{1}{301.7s}$$

(3) H(s)=0.0482

In the expression (2), Y(s) is the output level of the tank (T1). We change this name to avoid misconceptions with the gain of the signal conditioning circuit H(s). With the previous mathematical expressions in the Laplace domain, we simulated and compared these ones with the real data, employing MATLAB R2018b. Real and modeled data after performing these simulations are closely related.

### 8.2 Open loop modeling and simulation

**Fig. 27:**
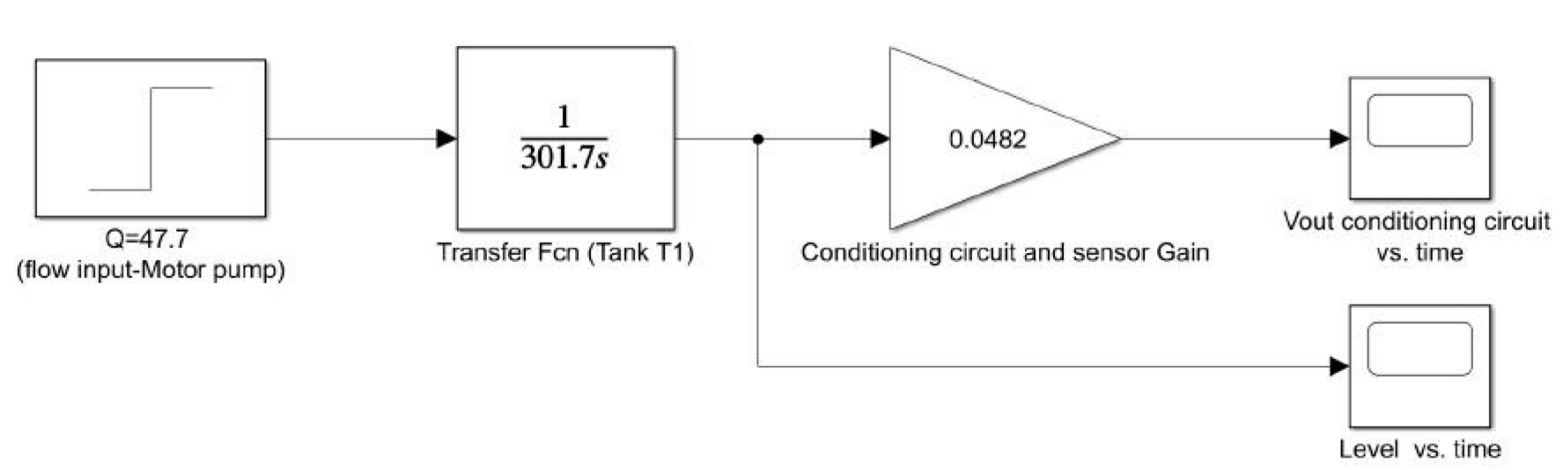
Open loop Simulink model for the tank T1 (control plant).

**Fig. 28:**
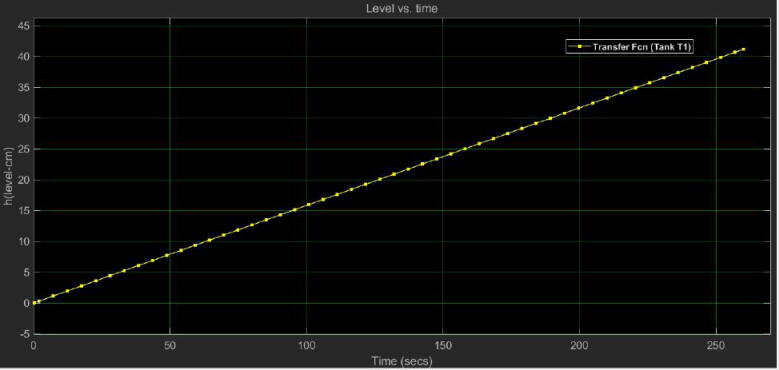
Open loop simulation for the transfer function of the tank T1 (control plant).

**Fig. 29:**
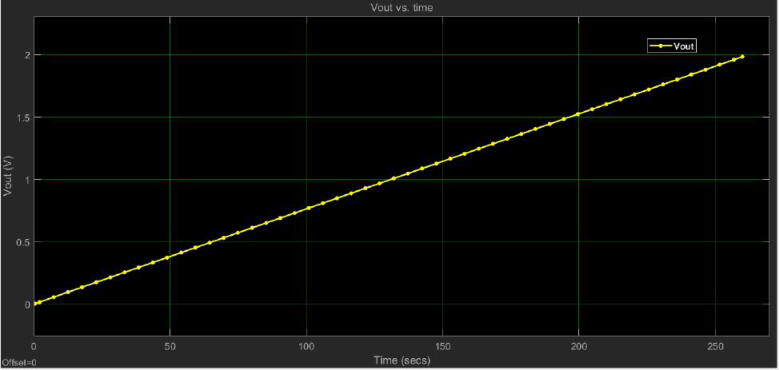
Simulation for the $V_{\mathrm{out}}$ of the conditioning circuit.

With the previous mathematical expressions in Laplace domain, we simulated their step response in Simulink from MATLAB. We emulated the flow (Q) of the motor pump as a step whose final value was $Q=47.77\frac{{\mathrm{cm}}^{3}}{\sec}$. Besides, we added a gain of 0.0482 for the sensor and the circuit of signal conditioning to get its output voltage $V_{\mathrm{out}}$ (see Fig. (28)). Fig. (27) depicts the Simulink model and the Fig. 29, Fig. 28 its simulation results.

Notice that in both plots the simulated behavior is similar to the experimental data presented in Fig. 25, Fig. 26), which corroborates that the mathematical models in the Laplace domain for the control plant and the conditioning circuit are suitable and match with the real data. With the Simulink model, the next step was the controller design and check the closed-loop response for the control plant.

### 8.3 Controller design

**Fig. 30:**
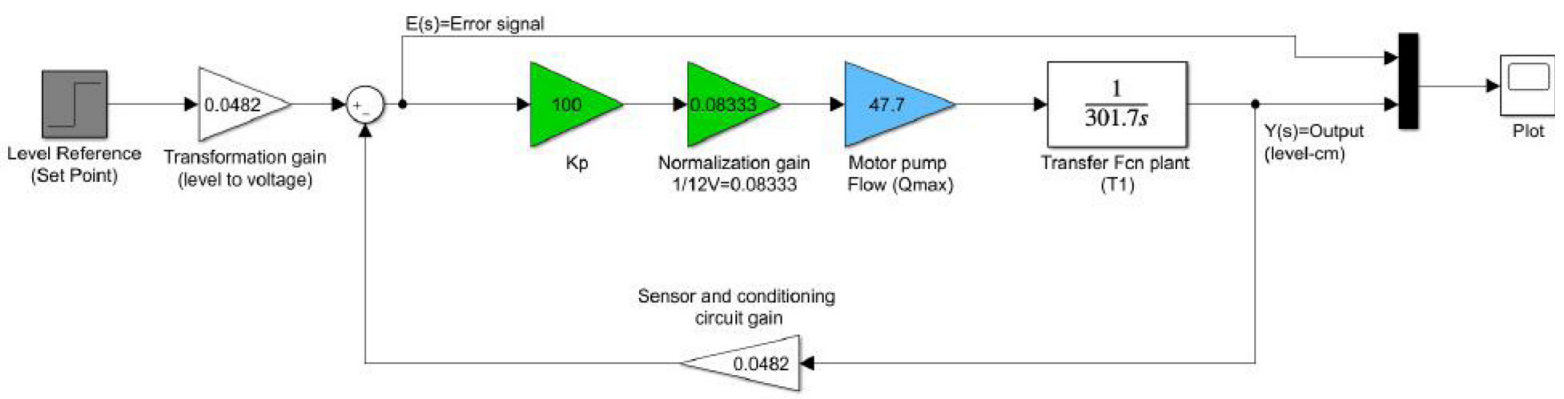
Closed-loop schematic for the plant and P controller.

For the controller design, we employed the Single-Input, Single-Output (SISO) Tool from MATLAB. Besides, we construct three classical controllers: Proportional (P), Proportional-Integral (PI), and Proportional-Integral-Derivative (PID) for the control system and we compared their performance. All controllers were implemented in both Arduino and in the web interface constructed for the RL in Python language. We used Arduino in the initial stages of development of the hardware and software components to check the stability of the controllers and agile the process of deployment of the control plant. Regarding the Proportional Controller (P), we started with an experimental Proportional Constant $K_{p}=30$. This value is the lower limit in which the plant did not respond to the controller compensation. After a progressive increase of $K_{p}$ from 30 to 100, we found this last value as the optimum for the response of the plant. Then, we simulated the closed-loop step response with the P controller in Simulink with the schematic illustrated in Fig. (30).

**Fig. 31:**
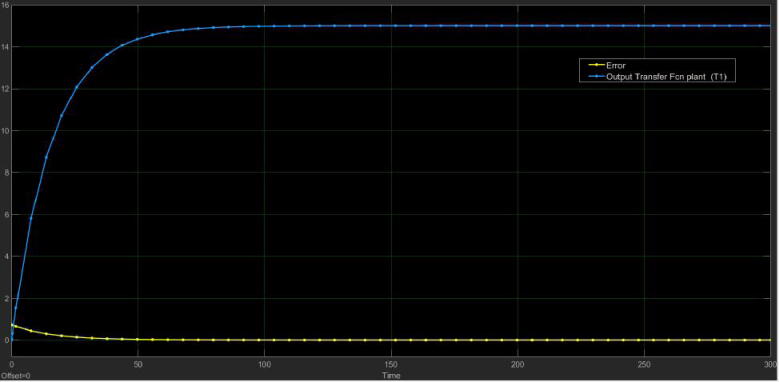
Closed-loop step response with P controller for a set point (h=15cm), $K_{p}=100$.

In green is represented the P controller with a normalization gain of 0.08333 which is equivalent to 1/12 V. This relation maps the output of the controller to a range between 0–12 V for the driver L298 because the motor pump works with these voltages, which are equivalent for the PWM signal to a duty cycle *k* in the range (0⩽*k*≤1). In blue appears the maximum flow $Q_{\max}$ of the motor pump found previously, while in gray is the set point (level desired) for the plant. Fig. (31) shows the closed-loop response for the plant with the P controller and a set point (h=15cm).

A similar procedure was performed for the PI and PID controllers. For these cases, we utilized directly SISO tool from MATLAB. As for the PI controller, we added an integrator and a real zero at the location −0.008333 to this tool, starting from the expression for a PI controller in the Laplace domain in the Eq. (4). The real zero was found after several iterations and comparations of the closed-loop response with the SISO tool. In the Eq. (4), $K_{p}$ is the proportional constant, and $K_{i}$ is the integral constant.

(4) $$C_{\mathrm{Pi}}(s)=K_{p}+\frac{K_{i}}{s}=\frac{K_{p}*(s+a)}{s},a=\frac{K_{i}}{K_{p}}$$

**Fig. 32:**
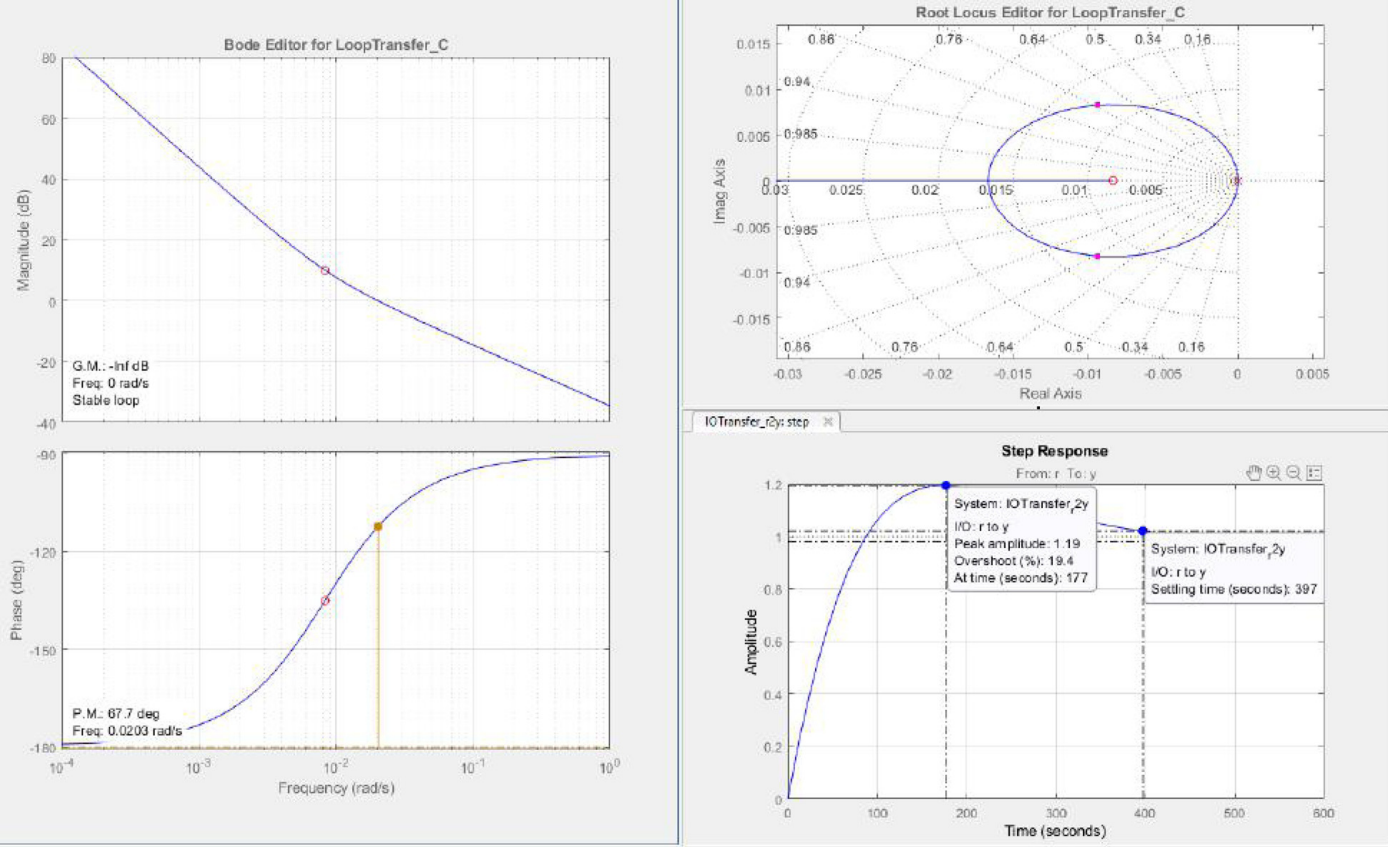
Closed-loop features with PI controller produced by SISO tool.

The SISO tool produced the expression in Eq. (5) with the previous parameters. The Bode, root locus, and step response plots illustrated in Fig. (32) show the system stability and an overshoot percentage of 19.4%.

(5) $$C_{\mathrm{Pi}}(s)=\frac{120*(s+0.008333)}{s},a=0.008333,K_{p}=120,K_{i}\approx 1$$

**Fig. 33:**
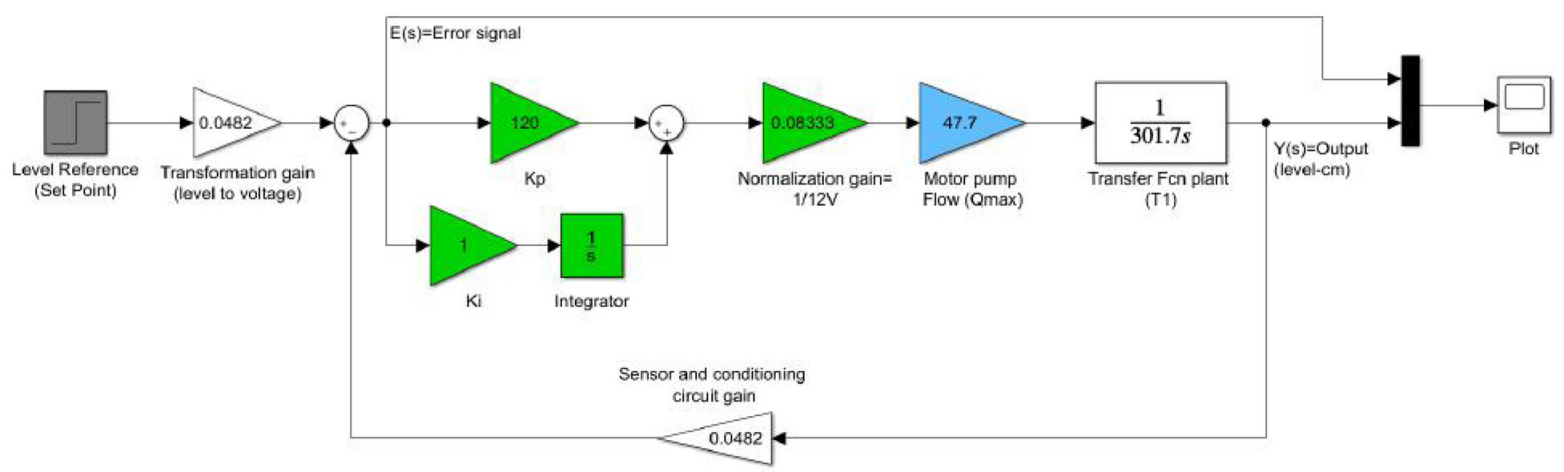
Closed-loop scheme with PI controller. $K_{p}=120,K_{i}=1$.

**Fig. 34:**
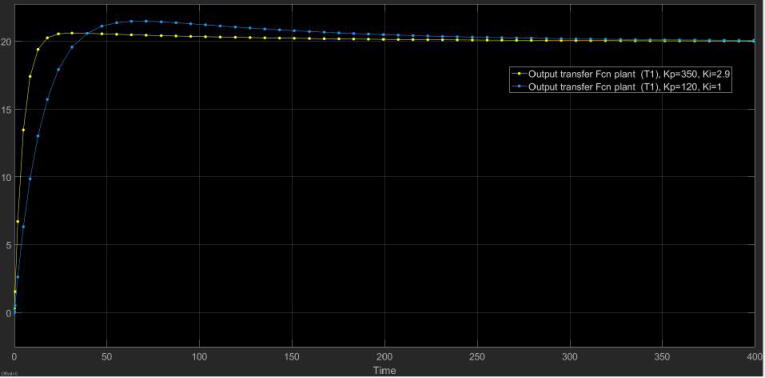
Plant step response with the PI controller, set point (h=20cm), and different values of $K_{p}$ and $K_{i}$.

However, previous values yielded a slower response of the plant and an overshoot of approximately 20%. To compensate this fact, we iterated until to find a set of optimum values for $K_{p}=350,K_{i}=2.9$. With the initial values of $K_{p}=120,K_{i}\approx 1$, we simulated the response of the plant according to the scheme in Fig. (33). Nonetheless, the experimental data with the values of $K_{p}$ and $K_{i}$ taken in the iterations are available in the dataset of the project in Section 4. (See Fig. (34))

Concerning the PID controller, we took the following expression for its design:

(6) $$C_{\mathrm{Pi}}(s)=K_{p}+\frac{K_{i}}{s}+K_{d}*s=\frac{K_{d}*(s+a)(s+b)}{s}$$

where $K_{d}$ is the derivative constant. Based on the previous values for the PI controller, we added a second real zero at the location −0.018 in the SISO tool, ensuring do not exceed an overshoot of 10%. This second real zero led to the following expression for the PID controller:

(7) $$C_{\mathrm{Pid}}(s)=\frac{6720*(s+0.083333)(s+0.018)}{s}$$

**Fig. 35:**
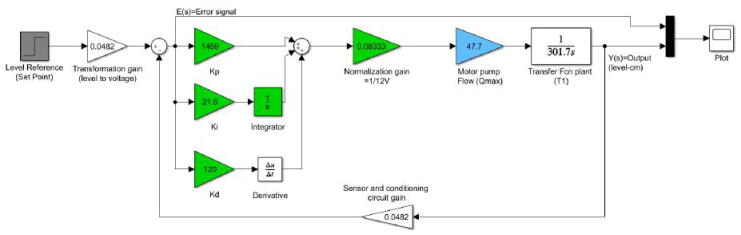
Closed-loop scheme with PID controller. $K_{p}=1459,K_{i}=21.6,K_{d}=120$.

**Fig. 36:**
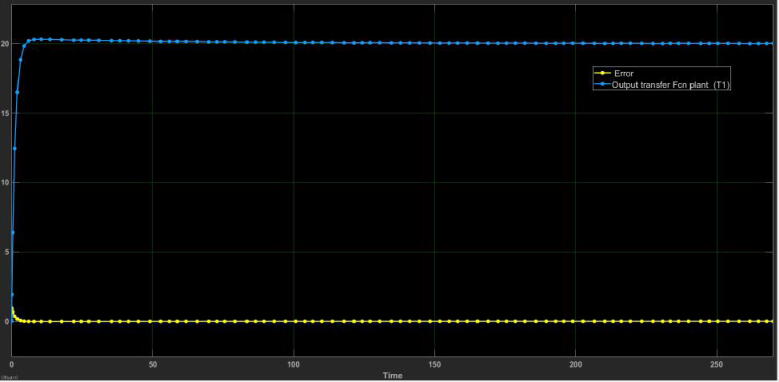
Plant step response with PID controller. Set point (h=20cm). $K_{p}=1459,K_{i}=21.6,K_{d}=120$.

Nevertheless, a value of $K_{d}=6720$ is very large and it is not suitable for implementation purposes. Thus, we selected a value of $K_{d}=120$ to test the PID controller and observe its response. This $K_{d}$ value yields to $K_{i}=21.6$ and $K_{p}=1459$. Fig. 35, Fig. 36 describe the closed-loop PID scheme and its step response with the previous values.

### 8.4 Controller implementation

For the implementation, each one of the previous controllers (P, PI, PID) in the Laplace domain were transformed into Z transform for their discretization. So, we employed the method Zero-Order Holder (ZOH) for this aim with a sampling time $T_{s}=0.6\mathrm{secs}$. Table (3) represents the Laplace domain and the Z transform equivalents for the controllers. In the table, [E(s),E(z)] are the control system errors, and [C(s),C(z)] are the outputs for the controllers in Laplace and *z* domains, respectively.

**Table 3:** Expressions for the controllers in Laplace domain (s) and *z* transform for the control tank system.

**Controller**	**Laplace domain (s)**	**Z Transform (z)**
Proportional Controller (P)	$\frac{C(s)}{E(s)}=100$	$\frac{C(z)}{E(z)}=100$
Proportional-Integral Controller (PI)	$\frac{C(s)}{E(s)}=120\;*(\frac{s+0.008333}{s})$	$\frac{C(z)}{E(z)}=\frac{120z-119.4}{z-1}$
Proportional-Integral-Derivative Controller (PID)	$\frac{C(s)}{E(s)}=\frac{130\;*(s+0.083333)(s+0.018)}{s}$	$\frac{C(z)}{E(z)}=\frac{7.754z^{2}-15.47z-7.716}{z^{2}-1}$

Once the controllers were transformed to the *z* domain, we found the respective difference equations for them in order to perform their implementation in Python. Table (4) shows the difference equations in each case. In the table, C(kT),E(kT) are the discretized output of the controller with its error, E(kT-1) is the previous sample of the error e(kT) in a sampling time $T_{s}$ before, E(kT-2) is the error two samples before, and so forth. The same applies to the output of the controller C(z).

**Table 4:** Expressions for the controllers in *z* transform and difference equations (kT) for the control tank system.

**Controller**	**Z Transform (***z***)**	**Difference Equation (***kT***)**
Proportional Controller (P)	$\frac{C(z)}{E(z)}=100$	C(z)=100*E(kT)
Proportional-Integral Controller (PI)	$\frac{C(z)}{E(z)}=\frac{120z-119.4}{z-1}$	C(kT)=120*E(kT)-119.4*E(kT-1)+C(kT-1)
Proportional-Integral-Derivative Controller (PID)	$\frac{C(z)}{E(z)}=\frac{7.754z^{2}-15.47z-7.716}{z^{2}-1}$	C(kT)=7.754*E(kT)-15.47*E(kT-1)+7.716*E(kT-2)+C(kT-2)

**Fig. 37:**
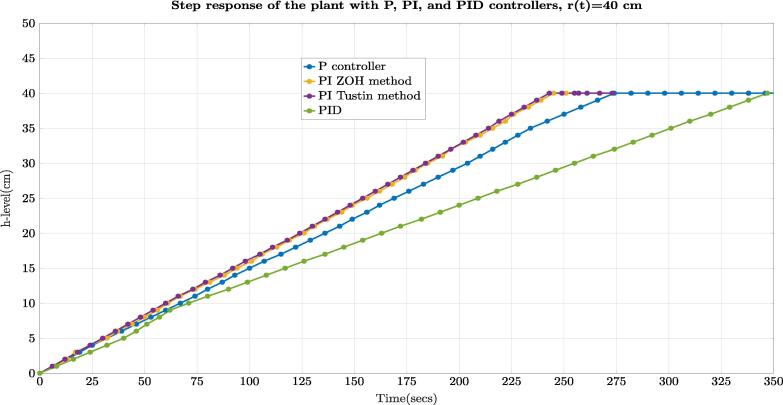
Experimental step response of the system under the P, PI, and PID controllers. Setpoint r(t)=40cm.

With the difference equations in Table (4), we implemented each controller in Python language, utilizing the web user interface. Algorithm.  (1) shows a sample of one script created for the PI controller with a setpoint r(t)=25cm. For instance, line 25 in the algorithm.  (1) shows the difference equation for this controller according to the expression of Table (4) with a change in the Proportional Constant of $K_{p}=350$ for better performance. Besides, lines 26–34 describe the limits (saturation-anti windup) for the controller with the PWM for the motor pump on GPIO 12. After, we compared the closed-loop step response and the settling time $t_{s}$ of each controller implementation for a given setpoint (r(t)=40cm) as Fig. (37) depicts. In this figure, the PID controller had the worst performance ($t_{s}\approx 337\mathrm{secs}$), while the PI controller with the ZOH method had the best performance ($t_{s}\approx 243\mathrm{secs}$). Similarly, the PI controller with the Tustin method (bilinear transform) obtained a similar performance to the PI with the ZOH method. Each one of the Python scripts for the controllers with their comments is available in the repository in Section 4.

**Table:** 

Listing 1: Python example of PI controller (ZOH method).
1: #Proporcional Integral Controller (PI) with ZOH (Zero Order Holder) method
2: import time
3: import board
4: import busio
5: import adafruit_ads1x15.ads1115 as ADS
6: import RPi.GPIO as GPIO
7: from adafruit_ads1x15.analog_in import AnalogIn
8:
9: Reference = 25.0 #Setpoint (desired level in cm)
10: kp = 350.0 #Proportional constant
11: PreviousError = 0.0 #Error variables
12: Error = 0.0
13: Sensor = 0.0
14: PreviousControl = 0.0
15: controlpi = 0.0
16: DutyCycle = 0.0
17: GPIO.setmode(GPIO.BCM)
18: GPIO.setup(13,GPIO.OUT)
19: pwm13 = GPIO.PWM(13,490) #GPIO 13 with PWM, f = 490 Hz
20: pwm13.start(0)
21: GPIO.setmode(GPIO.BCM)
22: GPIO.setup(12,GPIO.OUT)
23: pwm12 = GPIO.PWM(12,490) #GPIO 12 with PWM, f = 490 Hz
24: ts = 0.6 #Sample Time
25: ads = ADS.ADS1115(i2c) #Configure ADS1115
26: chan = AnalogIn(ads, ADS.P0) #Read ADS1115 channel 0.
27:
28: while True: #Infinite Loop
29: Sensor = round(chan.voltage,3) #Read voltage sensor output.
30: Error = round ((Reference*0.0482)-Sensor,3) #Calculate error
31: #control equation
32: controlpi = round (Error * kp - 119.4 * PreviousError + PreviousControl,3)
33: if (controlpi > 1.0):
34: controlpi = 1.0
35:
36: if (DutyCycle < 0.0): #(antiwind-up) PI control
37: DutyCycle = 0.0
38: if (Error < 0.0):
39: Error = 0.0
40: if (controlpi < 0.0):
41: controlpi = 0.0
42: DutyCycle = round (controlpi * 100,3)
43: pwm12.ChangeDutyCycle(DutyCycle) #PWM duty cycle depends on the PI Controller
44: time.sleep (ts)
45: PreviousControl = controlpi
46: PreviousError = Error

**Fig. 38:**
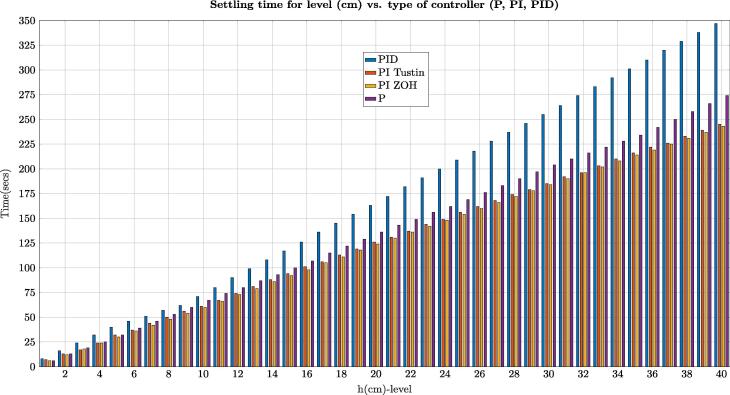
Experimental settling time ($t_{s}$) for each setpoint in the range (1 cm–40 cm) vs. type of controller (P, PI, PID).

Even though we described the results for a setpoint r(t)=40cm, we made several tests, starting from a set point of r(t)=1cm with the different controllers (P, PI, PID), which are illustrated in Fig. (38). In all experiments, the PID controller got the lower performance of all controllers implemented. The previous results demonstrate that the processes of identification, design and implementation of the controllers are congruent and suitable for the hardware and software created in this study.

### 8.5 RAM Memory consumption of the Raspberry Pi

**Fig. 39:**
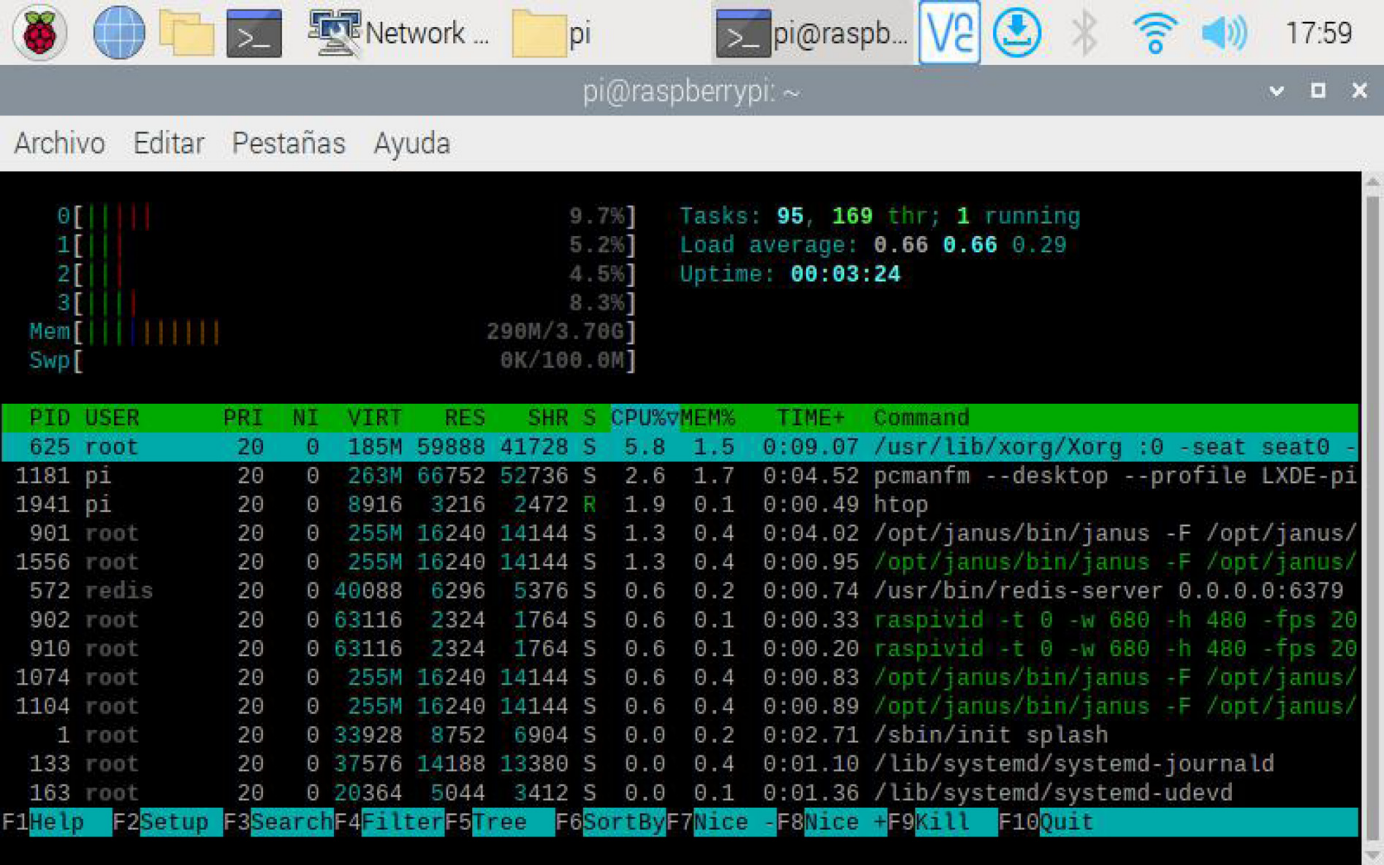
Results of *Htop* process viewer for the RAM consumption of *RaspyControl Lab*.

To finish the tests of the hardware designed, we checked the RAM consumption of the Raspberry Pi with the real-time video and the execution of the digital controllers (P, PI, PID), employing the tool *htop*[38] for Linux-based systems. Fig. (39) shows the results produced by this interactive process viewer. In this, the maximum RAM consumption was 290 MB from a 4 GB total memory of the Raspberry Pi with the main software components such as the Janus WebRTC server or the video streaming running (see green lines). This fact demonstrates that the experiment can run in a Raspberry Pi with 2 GB of RAM or less, which can help to reduce the costs of implementation and deployment of the experiment.

## 9 Conclusions and further work

In this article, we described the hardware and software components of the real-time remote laboratory *RaspyControl Lab*. We planned this laboratory to provide a high-quality and low-cost experimentation tool that any educator and student can build and employ to teach and learn automatic control systems, utilizing Python language. In general terms, the cost of *RaspyControl Lab* could oscillate between USD 420 to USD 461, changing the components mentioned in the section of the Bill of Materials (BOM). Besides, all hardware and software components are available in the repositories built for the project under the respective Creative Commons Attribution-ShareAlike license. The different validation experiments demonstrated that *RaspyControl Lab* is feasible and suitable to learn and experiment in control systems with few and low-cost hardware and software components. We hope that the software and hardware that we have created, help to bring the area of automatic control systems closer to students and stakeholders who wish to learn and experiment much more about the concepts of this area. Further work will be focused on creating new real-time experiments for automatic control and programming using Python language, starting from the software and hardware created in this study.

## CRediT authorship contribution statement

**Jonathan Álvarez Ariza:** Conceptualization, Formal analysis, Supervision, Software, Writing - original draft, Writing - review & editing. **Christian Nomesqui Galvis:** Investigation, Formal analysis, Validation.

## Declaration of Competing Interest

The authors declare that they have no known competing financial interests or personal relationships that could have appeared to influence the work reported in this paper.
